# Depression’s double-edged impact on body mass index. A hidden catalyst for non-communicable diseases in South Africa’s aging population in long-term care facilities

**DOI:** 10.1371/journal.pone.0319188

**Published:** 2025-02-13

**Authors:** Shane Naidoo, Nirmala Naidoo

**Affiliations:** Department of Health and Rehabilitation Sciences, University of Cape Town, Cape Town, South Africa; The University of New Mexico, UNITED STATES OF AMERICA

## Abstract

**Introduction:**

The global population of older people, who have a high prevalence of non-communicable diseases, is on an upward trajectory, notably in South Africa. The expansion of this demographic will further strain an already overwhelmed healthcare system, primarily taxed by infectious diseases in younger populations. Physical activity has been shown to effectively reduce risk factors for non-communicable diseases in older people.

**Objective:**

To investigate the associations between depression, body mass index (BMI) and physical activity and its complex interplay on non-communicable diseases in older people residing in South African long-term care facilities.

**Method:**

We conducted a cross-sectional study on 396 participants residing in South African rural and urban long-term care facilities to analyse BMI, waist-hip ratio, physical activity and depression levels. The Geriatric Depression Scale and the International Physical Activity Questionnaire was used to evaluate depression and physical activity respectively.

**Results:**

The sample had a mean BMI of 27.53 kg/m^2^ (95% CI [26.99, 28.07], SD = 5.49), with an obesity prevalence of 31.82%. Additionally, 35.10% of participants exhibited some degree of depressive symptoms. In an ordinal regression model BMI was a significant predictor (*B* = .10, *p* = .007) for increases in depression. Conversely in a linear regression model, depression (*B* = -2.01, *p* = .004) and physical activity (*B* = -.001, *p* = .008) were predictors for decreases in BMI.

**Conclusion:**

The relationship between depression and BMI in older people is complex, with depression often leading to both weight loss and, conversely, increased BMI contributing to a higher risk of depression. Physical activity serves as a critical intervention, helping to reduce both BMI and depressive symptoms among older people residing in long-term care facilities. This underscores the importance of integrating PA programs into care strategies to improve overall health and well-being in this population.

## 1 Introduction

### 1.1 Background

In South Africa, depression accounts for an estimated 4.5–5.5% of the total Years Lived with Disability (YLD) in individuals over 60 years old. This mirrors global evidence identifying depression as a subtle yet profound catalyst that significantly exacerbates the risk of disability in the aging population [1]. Investigations into the elderly population in South Africa demonstrate a marked prevalence of depression, with rates peaking at 37.3% in long-term care facilities (LTCF) and increasing to 40–45% among community-dwelling individuals [2–4]. South Africa’s escalating elderly population, has a high prevalence of non-communicable diseases (NCDs), often presenting as comorbidities. Hypertension, with an incidence ranging from 63.7% to 68.8%, stands as the most prevalent condition among older people in LTCFs. Joint disorders, affecting 38.2% to 46.2% of this population, follow closely, while heart disease is reported in 22.1% to 32.4% of individuals. Notably, depression is prevalent in 37.3% of older people, underscoring these four conditions as the most frequently documented comorbidities in this demographic [4, 5]. These comorbidities closely mirror the chronic conditions observed in older people from developed nations residing in LTCFs [6, 7]. However, the extent of these comorbidities may vary considerably between developed and developing nations, shaped by disparities in healthcare infrastructure, differential socioeconomic determinants, and the unique health profiles of older people [8]. Non-communicable diseases and depression in older people are intricately linked in a bidirectional manner, where each can influence the onset, progression, and severity of the other [3]. Extreme BMI (obesity or malnutrition) and physical inactivity are cited as two of the risk factors that are critically implicated in the aetiology and progression of NCDs among older people [9–11]. Depression fosters obesity by reducing physical activity (PA) and promoting unhealthy eating patterns, while obesity further diminishes activity due to mobility challenges and increased discomfort [3]. This cyclical interaction between depression, obesity, and physical inactivity exacerbates the health risks associated with NCDs. The exploration of the interrelations between depression, nutritional imbalance, and PA among older people in South African LTCFs is limited.

### 1.2 Objective

With the rapid growth of this demographic and the high incidence of NCDs, the objective of this study was to explore potential associations between these risk factors in older people residing in South African LTCF. Understanding these correlations is vital for implementing targeted strategies to mitigate the health risks associated with NCDs and improve the quality of life for older people in these settings.

## 2 Methods

### 2.1 Study design

This study followed a cross-sectional design.

### 2.2 Research setting

The sample was drawn from the eThekwini municipality, KwaZulu-Natal province, South Africa. The eThekwini municipality, South Africa’s third most populous, has a total population of 3.9 million, including the fifth largest proportion (4.8%) of older people among metropolitan areas [12]. eThekwini Municipality differs from other major metropolitan municipalities as 68% of the metropole is classified as rural and almost one quarter of the population live in informal establishments [13].

### 2.3 Participants

Participants in this study were older people (≥ 60 years), regardless of biological sex, residing in LTCFs within the eThekwini municipality, and who had ability to give informed consent. All prospective participants were screened by the researcher, a qualified physiotherapist for inclusion. Participants were excluded if:

- The study procedures were deemed potentially harmful as participants did not understand testing instructions.
- Failed the Integrated Care for Older People (ICOPE) screening tool’s cognition and hearing components [14]. This is a simple screening tool, designed by the World Health Organisation, that detects motor, cognitive, nutritional, vision, hearing and psychological intrinsic declines in older people (S1 Appendix).
- Participation in other studies/investigations.
- Medication that altered their cognition or affected their performance in the testing procedures.
- Participants were currently under treatment for an acute illness (influenza, pneumonia, COVID-19) or recently recovered (< seven days), from an acute illness.
- Resting heart rate greater than 100 beats per minute and/or resting oxygen saturation less than 96% [15].
- A resting systolic blood pressure ≥ 139 mmHg and/or resting diastolic blood pressure ≥ 99 mmHg [16].

### 2.4 Sample and recruitment

Epi Info^TM^ Version 3 [17], was used to calculate the sample size using 50% prevalence of NCD in older people as reported by Wu et al. [9]. A sample size of 384 was determined to be requisite, using a 95% confidence interval for statistical robustness. A sample of 400 was recruited to allow for attrition. Rural and urban areas in the eThekwini municipality were the study settings with research showing that 68% of the population live in urban areas [18]. Focusing on LTCF residents, we stratified our sample based on the distribution of the population between urban and rural areas. Consequently, we randomly selected seven urban and three rural LTCFs to reflect this distribution with 40 randomly selected participants from each facility to minimise biases.

### 2.5 Ethical considerations

Ethics clearance was granted and permission to conduct the research in the province was obtained from the KwaZulu-Natal Department of Social Services, with consent also attained from the respective randomly selected LTCFs. Participant recruitment took place from September 18 to 29, 2023, followed by the screening process in October 2023. A total of 396 older people who satisfied the inclusion criteria and who provided written signed informed consent, were subsequently enrolled in the study.

### 2.6 Outcome measurements

Demographic characteristics, including chronological age, biological sex, level of educational attainment, and marital status, were documented (S2 Appendix). To address the research objectives, this study focused on a comprehensive set of measurement outcomes that reflect the multifaceted nature of health in older people. Body mass index (BMI) was calculated using anthropometric measures of height (m) and weight (kg). Its value gives a broad perspective on the nutritional status of an adult and classified as outlined in Table 1 [19].

**Table 1:** WHO BMI classification for adults.

BMI (kg/m^2^)	Nutritional status
Below 18.5	Underweight
18.5–24.9	Normal weight
25.0–29.9	Pre-obesity or overweight
30.0–34.9	Obesity class I
35.0–39.9	Obesity class II
Above 40	Obesity class III

BMI: body mass index, WHO: World Health Organisation

Handgrip strength (HGS) is a good indicator for detection of health status decline in older people [20]. It provides an overall indication of general muscle strength (Table 2), and declines in HGS are strong indicators of frailty [21].

**Table 2:** Classification of hand grip strength.

Age (Years)	Males HGS (kg)			Females HGS (kg)		
	Weak	Normal	Strong	Weak	Normal	Strong
**60–64**	30.20	30.20–48	> 48	17.20	17.20–31	> 31
**65–69**	28.20	28.20–44	> 44	15.40	15.40–27.20	> 27.2
**70–99**	21.30	21.30–35.10	> 35.10	14.70	14.70–24.50	> 24.5

HGS: handgrip strength

Physical activity was assessed using the International Physical Activity Questionnaire–Short Form (IPAQ-S). The IPAQ-S is a valid and reliable tool to assess PA in older people in a South African context and takes few minutes to complete [22]. It is a self-reported tool capturing PA of at least ten minutes across three intensity levels, (walking, vigorous, and moderate) over the past seven days [22]. These activities are then converted into Metabolic Equivalent of Task minutes per week (MET min/week), quantifying energy expenditure (Table 3). Sedentary activity, measured in weekday minutes (maximum 180), was extracted from the IPAQ-S as an outcome metric.

**Table 3:** Classification of PA levels from IPAQ-S scoring MET min/week.

Low levels of PA	No activity or MET min/week < 600
Moderate levels of PA	MET min/week ≥ 600 but < 3000
High levels of PA	MET min/week ≥ 3000

MET min/week: Metabolic Equivalent of Task minutes per week, PA: physical activity, IPAQ-S: International Physical Activity Questionnaire

Waist-hip ratio is a measure of waist circumference (cm) divided by hip circumference (cm) [23]. This measure is a good comparative when testing for overweight/obesity as BMI measurements may overlook abdominal adiposity [23]. The waist-hip ratio predicts the risk of metabolic complications for values ≥ .9 in males and ≥ .85 in females [23]. The Barriers to Being Active Quiz uses a 4-point Likert scale (0 being unlikely and 3 being very likely) to measure self-reported barriers to PA in seven categories (lack of time, social influences, lack of energy, lack of willpower, fear of injury, lack of skill, and lack of re-sources) [24]. Each category has three questions which in total are scored from zero to nine. A score of five or greater in any category indicates a PA barrier.

The Short Physical Performance Battery (SPPB) is subdivided into tests for gait speed, balance, and chair sit-to-stand. The three tests are each ranked with a score between 0 to 4 which are added to attain a total score. Maximum score achievable is 12, with zero being minimum score. The SPPB is a reliable and valid means of identifying disability [25], predicting risks of falling [26], and decline in mobility and function [27]. The Weighted Functional Comorbidity Index comprises of 18 weighted comorbidities questions with severity rating for each of the conditions (Table 4). Total index scores range from 0–36 with severity of functional impairment directly correlated with the Comorbidity Index score. The questionnaire documented self-reported comorbidities, quantified as an outcome measure.

**Table 4:** Rating scale for functional severity [28].

Rating	Severity of Comorbidity
**0**	Disease is present with no influence
**1**	Disease present partly causing functional impairment
**2**	Disease present causing severe functional impairment

The Geriatric Depression Scale–Short Form (GDS) was utilised to evaluate depression levels. The GDS was selected based on its validated use in older people studies within South Africa [2, 29], its relevance to LTCF residents [30], and its suitability for older people with mild cognitive impairment or physical frailty [31]. This concise 15-item questionnaire is completed within 5 minutes [31]. Ten items signal depression with positive responses, while five indicate depression with negative responses. Scores range from 0 to 15, with 0–4 suggesting no depression, 5–8 indicating mild depression, 9–11 signifying moderate depression, and 12–15 denoting severe depression.

The EuroQol 5-Dimension 3-Level (EQ-5D-3L) questionnaire has two parts, a descriptive section (EQ-5D) and a visual analogue scale (EQ-5D-3L VAS). The EQ-5D has five constituents (self-care, anxiety/depression, mobility, pain/discomfort, and usual activities) with three levels for each constituent (no problems, some problems, and extreme problems) [32]. The EQ-5D-3L index is tallied by subtracting the total of the descriptive section from the numerical value 1. An index score of 1 represents the best possible health status, and values < 0 indicates the worst possible health status [33]. The EQ-5D-3L VAS allows participants to indicate their perceived health status using a visual analogue scale that ranges from 0 to 100%.

### 2.7 Statistical analysis

Statistical analysis utilising SPSS Version 27 was used to analyse the data. Tests for normality was undertaken and descriptive statistics used. Data that was normally distributed was analysed with t -tests to check for differences between groups. One-way ANOVA tests was used to test the relationship between normally distributed ratio/continuous data and multiple level categorical variables. Data that was not distributed normally was analysed with the Mann-Whitney U Test (Wilcoxon Rank-Sum test). The Kruskal-Wallis H test was used to test the relationship between ratio/continuous data that was not normally distributed and multiple level categorical data. Chi-squared analyses was used to check for differences in categorical data. Pearson’s correlation tests were used to analyse relationships between parametric data, with Spearman’s correlation tests used for non-parametric data. The strength of the Pearsons (r), and Spearman’s rho (ρ) correlation coefficients were analysed according to the correlation scale.

To investigate the predictive relationships of variables, we employed different regression models tailored to the nature of the dependent variables. A multiple regression linear analysis was conducted to examine the predictors of BMI. Dummy variables were created for all categorical values. The regression model ran BMI as dependant variable on independent variables (backward selection) of age, biological sex, education, systolic blood pressure, diastolic blood pressure, resting heart rate, oxygen saturation, HGS, waist-hip ratio, MET min/week (vigorous, moderate, walking), sedentary minutes, SPPB scores, hip circumference, waist circumference, GDS, EQ-5D-3L and number of comorbidities. Ordinal regression analysis was conducted to examine the predictors of GDS scores. The predictor variables were biological sex, age, marital status, educational level, heart rate, systolic blood pressure, diastolic blood pressure, oxygen saturation, BMI, hip circumference, waist circumference, waist-hip ratio, HGS, sedentary minutes, EQ-5D-3L (VAS and index score), SPPB, EQ-5D-3L, number of comorbidities, vigorous, moderate, walking and total MET min/week.

## 3 Results

Among the 438 consent forms collected, 38 prospective participants did not arrive for testing. The researcher screened all participants using the ICOPE screening tool, excluding those with cognitive disorientation (*n* = 4), severe malnutrition, visual impairments, or failed hearing tests. These individuals were referred for medical follow-up. The final sample comprised of 396 participants, randomly selected from 17 LTCFs, with 22% residing in rural areas. Notably, 59.5% failed the three-word recall test, indicating short-term memory deficits, while 19.8% reported depressive symptoms on the ICOPE screening tool (Table 5).

**Table 5:** ICOPE screening positive participant’s responses.

ICOPE Screening		Urban		Rural	
		Females (%)	Males (%)	Females (%)	Males (%)
**Cognition**	Orientation to date and place	210 (100)	99 (100)	46 (100)	41 (100)
	Recalls three easy words correctly	85 (40.50)	42 (42.40)	14 (30.40)	19 (46.30)
**Mobility**	5 successful unassisted chair stands in 14 seconds	179 (85.20)	86 (86.90)	37 (80.40)	32 (78)
**Malnutrition**	Have you unintentionally lost > 3kg in the last 3 months.	10 (4.80)	2 (2)	2 (4.30)	1 (2.40)
	Have you lost your appetite?	28 (13.30)	5 (5.10)	6 (13)	4 (9.80)
**Vision**	Do you have problems with your eyes, difficulties in seeing far, reading, eye disease or currently under medical treatment (e.g. diabetes or high blood pressure)	146 (69.50)	56 (56.60)	25 (54.30)	22 (53.70)
**Hearing**	Pass whisper test	210 (100)	99 (100)	46 (100)	41 (100)
**Depression**	Over the last 2 weeks have you been bothered by–feeling down or depressed.	36 (17.10)	14 (14.10)	11 (23.90)	6 (14.60)
	Little interest in doing things.	47 (22.40)	22 (22.10)	13 (28.30)	4 (9.80)

Total *n* = 396: Urban female *n* = 210, Rural female *n* = 46, Urban male *n* = 99, Rural male *n* = 41

The sample had a mean age of 72.87 (95% CI [72.11,73.63], SD = 7.70) years, which was predominantly composed of females (*n* = 256; S3 Appendix). The mean BMI of the sample was 27.53 kg/m^2^ (95% CI [26.99, 28.07], SD = 5.49). Obesity rates were notably higher in urban areas, with females at 35.72% and males at 29.29%, compared to rural areas, where the rates were 26.08% for females and 24.59% for males. In contrast, overweight prevalence was more prominent among rural participants (47.55%) compared to their urban counterparts (28.88%; Table 6).

**Table 6:** Summary of participant demographics and outcome measures.

		Urban			Rural		
		Female	Male	Total	Female	Male	Total
Age (years)	Mean	72.54	71.65	72.26	77.24	72.59	75.05
Resting heart rate (bpm)	Mean	78.89	78.07	78.63	81.04	78.41	79.80
SBP (mmHg)	Mean	130.48	127.71	129.59	132.61	126.32	129.64
DBP (mmHg)	Mean	76.24	77.05	76.50	80.98	77.80	79.48
BMI (kg/m^2^)	Mean	27.88	26.72	27.51	27.77	27.40	27.59
Weight status (*n*)	Underweight	6	1	7	2	1	3
	Normal	65	42	107	16	11	27
	Overweight	64	27	91	16	19	35
	Obese	75	29	104	12	10	22
GDS total score	Median	3	3	3	4	4	4
GDS depression scale (*n*)	Nil	145	64	209	26	22	48
	Mild	48	25	73	15	17	32
	Moderate	15	9	24	5	1	6
	Severe	2	1	3	0	1	1
Waist-hip ratio	Mean	.91	.99	.93	.91	.94	.92
MET min/week (mean)	Vigorous	209.33	124.65	182.20	126.09	275.12	196.32
	Moderate	476.19	422.06	458.85	292.61	222.93	259.77
	Walking	1094.58	1214.67	1133.05	1007.93	991.61	1000.24
	Total	1693.95	1674.73	1687.79	1401.72	1267.22	1338.33
Sedentary minutes	Mean	151.64	153.54	152.25	150.43	157.56	153.79
HGS (kg)	Mean	14.46	19.90	16.20	12.85	24.19	18.19
SPPB total	Median	7	7	7	6	7	6
EQ-5D-3L VAS	Mean	70.30	71.52	70.69	69.78	69.51	69.66
EQ-5D-3L index	Mean	.82	.85	.83	.83	.86	.84
w-FCI score	Median	4	3	4	4	3	3
Number of comorbidities	Median	3	3	3	3	2	3

Total *n* = 396: Urban female *n* = 210, Rural female *n* = 46, Urban male *n* = 99, Rural male *n* = 41, SBP: systolic blood pressure, DBP: diastolic blood pressure, GDS: Geriatric Depression Scale, HGS: Hand grip strength, w-FCI: weighted Functional Comorbidity Index

**Fig 1:**
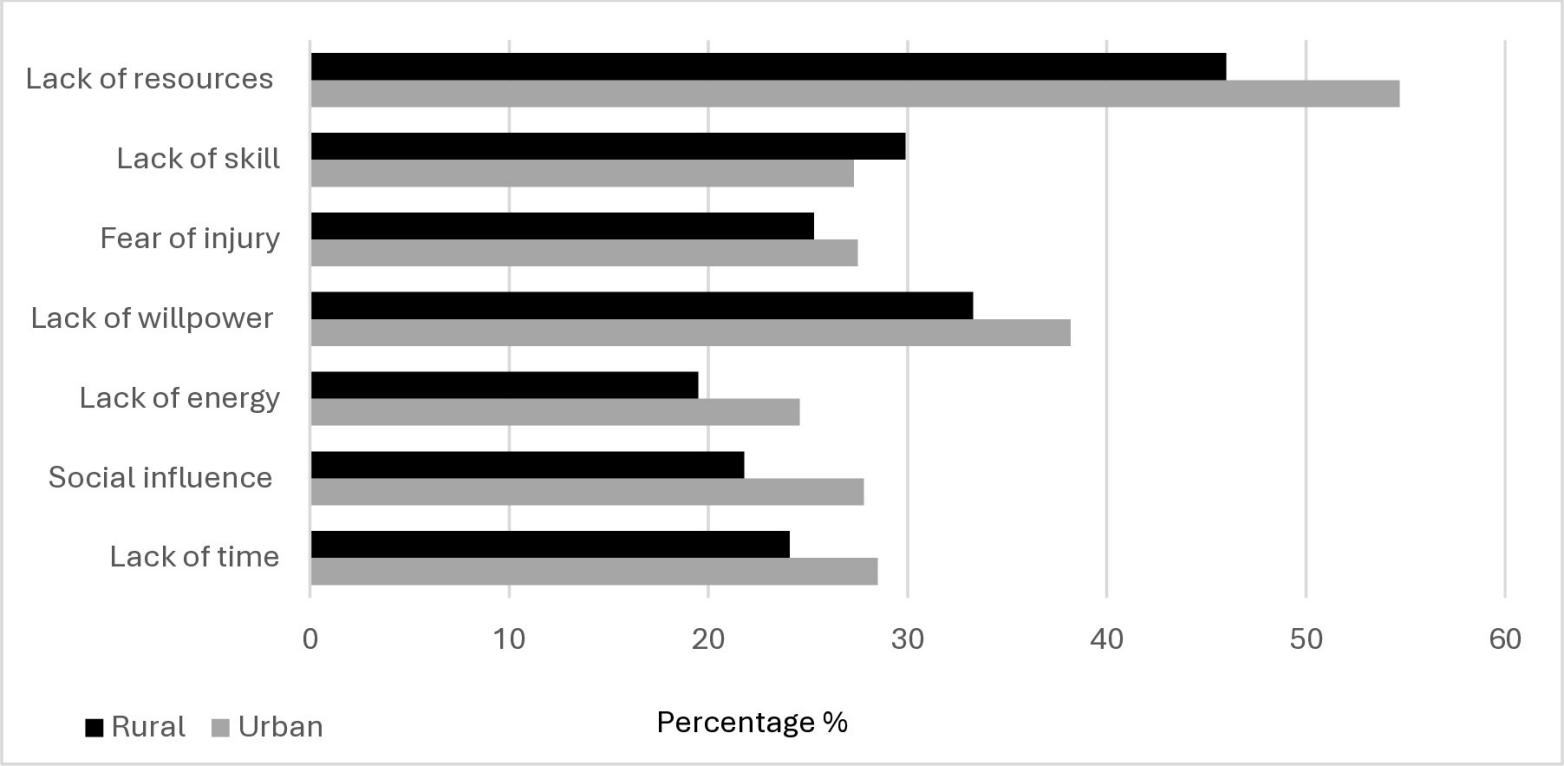
Barriers to Being Active Quiz participant responses.

Lack of resources was the highest PA barrier, cited by 54.20% of urban and 46% of rural participants (Fig 1).

The linear regression model for BMI was significant, *F*(12,383) = 6.99, *p* < .001, and accounted for approximately 18% of the variance in BMI (Table 7; S3 Appendix). The effect size (*f*^2^ = .22) suggested a moderate effect, indicating that the predictors had a meaningful influence on BMI. Geriatric Depression Scale had a negative unstandardised coefficient (*β* = −2.01), indicating an inverse relationship with BMI. This suggests that for every one-unit increase in the GDS score (indicating higher depression severity), BMI decreased by 2.01 kg/m^2^, holding other variables constant.

**Table 7:** Regression model coefficients.

Model	Unstandardised Coefficients		*t*–value	*p*–value
	β	Std. error		
**Constant**	17.97	4.87	3.69	**< .001**
**SPPB (balance)**	2.86	.75	3.83	**< .001**
**Systolic blood pressure**	.08	.03	2.75	**.006**
**GDS**	-2.01	.69	-2.91	**.004**
**Age**	-.10	.03	-3.04	**.003**
**w-FCI**	2.81	1.19	2.37	**.018**
**Hip-waist ratio**	5.77	2.10	2.74	**.006**
**Walking MET min/week**	.00	.00	-2.66	**.008**
**Sedentary minutes**	.01	.01	2.23	**.026**
**Arthritis**	.79	.34	2.34	**.020**
**BBAQ (lack of resources)**	2.67	1.15	2.33	**.020**
**BBAQ (lack of willpower)**	-1.76	.83	-2.11	**.035**
**SPPB total score**	-3.76	1.96	-1.92	.055

SPPB: Short Physical Performance Battery, GDS: Geriatric Depression Scale, w-FCI: Weighted Functional Comorbidity Index, BBAQ: Barriers to Being Active Quiz, MET min/week: Metabolic Equivalent of Task minutes per week

In addition to the regression analysis, the Kolmogorov-Smirnov test was conducted to assess the normality of the residuals. The test indicated no significant evidence that the residuals deviated from normality, *D* = .04, *p* = .49, with the Durbin-Watson (2.00) indicating that the residuals were independent. The Breusch-Pagan test was conducted to assess the presence of heteroscedasticity in the residuals, which showed no significant evidence of heteroscedasticity (χ^2^ = .04, *p* = .84).

The ordinal regression model for GDS was found to be statistically significant χ2(158) = 379.88, *p* < .001, and explained 62.3% (Pseudo- Nagelkerke *R*^2^) of the variance in GDS (Table 8; S3 Appendix). Variance Inflation Factor (VIF) checks were performed to assess multicollinearity among the predictor variables. All predictors exhibited VIF values below 10, indicating the absence of multicollinearity. This ensures that the estimated coefficients were stable, reliable, and not distorted by intercorrelations among the variables. BMI had a positive estimate, (*B* = .10), indicating a direct relationship with GDS. This suggests that as BMI increases, the likelihood of a higher GDS score (indicating greater depression severity) also increases. Specifically, for every one-unit increase in BMI, the odds of being in a higher GDS category increase by approximately 10%, holding other predictors constant.

**Table 8:** Predictors in ordinal regression model for GDS.

Predictor	Estimate (B)	SE	Wald χ^2^	*p*-value	95% CI (Lower)	95% CI (Upper)
SPPB total score	3.73	1.27	8.51	**.004**	1.22	6.24
Number of comorbidities	-8.86	3.55	6.21	**.013**	-15.82	-1.89
Body mass index	.10	.03	7.25	**.007**	.02	.17
Sedentary minutes	.006	.003	5.23	**.020**	.001	.01
EQ-5D-3L VAS	-.02	.007	12.05	**< .001**	-.04	-.01
Biological Sex (1-male, 2 -female)	-.82	.29	7.98	**.005**	-1.40	-.25

SPPB: Short Physical Performance Battery, EQ-5D-3L-VAS: EuroQol 5-Dimension 3-Level Visual Analogue Scale, GDS: Geriatric Depression Scale

## 4 Discussion

### 4.1 Demographics

The participants in this study predominantly comprised of females residing in LTCFs. Likewise, an independent investigation spanning 14 urban LTCFs within the eThekwini municipality reported a similar female predominance (73%), with a mean age closely mirroring that of our cohort [34, 35]. These statistics are consistent with the General Household Survey conducted in South Africa, which indicated that sixty percent of older people are women, corroborating the trend that women generally outlive men [36].

### 4.2 Integrated care of older people screening

The ICOPE screening application, to our knowledge, has not been used in a South African/African context prior to this study. The ICOPE screening tool was utilised to evaluate participant’s eligibility for inclusion in the study. During informational sessions held prior to testing, prospective participants were clearly informed that meeting specific eligibility criteria was mandatory for participation. As a result, only four prospective participants with cognitive impairments related to orientation to place, date, or time were excluded from the study based on the ICOPE screening tool assessment. A systematic review of studies from China, Taiwan, England, and India, utilising the ICOPE screening tool, determined the pooled prevalence of cognitive decline among older people to be 29.7% [37]. The prevalence of cognitive decline observed in our study was markedly higher, with 60% of participants demonstrating impairments, predominantly among rural-dwelling females. Kobayashi et al. [38], in their study of older people in rural South Africa, identified an inverse relationship between cognitive decline and age, as well as a positive association with educational attainment. The lower levels of formal education among rural participants in our study could have likely contributed to the increased prevalence of cognitive decline. This is further supported by an independent investigation conducted in urban LTCFs within the eThekwini metropole, which similarly reported a negative correlation between educational attainment and cognitive decline in older people [39].

Peltzer et al. [40], analysed data from the SAGE study on older people conducted in South Africa, reported that rural residing participants were at greater risk of cognitive decline than their urban residing peers. These findings align with those of an independent study conducted in another upper-middle-income country, which observed lower cognitive performance among rural older people compared to their urban counterparts, further corroborating the results of our study [41]. This comparison underscores the existence of geographic disparities in cognitive decline, indicating that rural settings may offer fewer cognitive health resources or present unique challenges not as prevalent in urban areas. These observations emphasise the importance of incorporating environmental and socioeconomic factors into strategies for assessing and addressing cognitive decline on a global scale.

In the mobility component of the ICOPE screening application, 13.95% of urban and 20.81% of rural residing older people, (total-17.38%), had difficulty in completing the test. These findings are consistent with a systematic review, which reported that 17.5% of older people failed the mobility screening assessment [37]. Furthermore, a comprehensive systematic review investigating sarcopenia among older people in urban and rural settings revealed that those residing in rural areas are at significantly greater risk of muscle loss and impaired mobility [42]. Similarly, our study demonstrated that older people residing in rural areas encountered greater challenges in completing the mobility screening compared to their urban counterparts. Factors such as the built environment, suboptimal nutrition, insufficient PA, and polypharmacy have been identified as key contributors to this disparity [42]. The ICOPE screening for malnutrition and appetite conducted in our study revealed a 6.84% prevalence of vitality loss, which is notably lower than the 8.5% combined prevalence reported by Jayaraj et al. [37].

The ICOPE vision screening component incorporated questions regarding participant’s management of diabetes and hypertension, in addition to vision-specific queries. This combined approach may have contributed to the high positive response rate of 58.53% observed for this component. However, assessing vision in older people on treatment for diabetes/hypertension is important for detecting unrecognized vision deterioration [35]. All participants that were hypertensive/diabetic and who reported no visual difficulty were referred for further medical investigation. A systematic review of the ICOPE screening tool found a combined 17.9% prevalence of visual decline [37], with the highest of 49% recorded in India [43]. Mashige et al. [35], reported a 63.6% prevalence of visual impairment and blindness in older people residing in eThekwini LTCFs. All subjects in our study had to pass the hearing test for inclusion so the screening in this component was 100% for the whisper test. Within our sample, depression prevalence, as determined through the ICOPE screening tool, was 19.04%, with the highest incidence recorded among rural women at 26.1%. A systematic review of studies utilising the ICOPE screening tool reported a consolidated depression prevalence of 17.9%, aligning closely with our observations.

### 4.3 Physical activity

Our sample revealed a general decline in PA with advancing age, consistent with findings from other studies of older people in LTCFs [44–46]. However, the total PA levels observed in our study were notably lower than those reported elsewhere [44, 46]. Rural participants in our study had a lower mean PA level as compared to urban participants. No study in LTCF was available to confer these findings with the closest contextual study comparing PA between urban and rural community residing older people. Community-dwelling older people tend to engage in greater levels of PA compared to their counterparts in LTCFs [44]. Moreover, older people residing in rural areas generally demonstrate higher PA levels than those living in urban environments [47]. We observed the converse in our participants and some of the probable reasons for this disparity could attributed to resource constraints [48], staff turnover and shortages [49], geographical isolation [50], and cultural perceptions [51].

### 4.4 Body mass index

Our findings indicated that urban-dwelling participants exhibited a higher prevalence of obesity compared to their rural counterparts. However, the difference in mean BMI between the two groups was minimal and not statistically significant according to the *t*-tests. One of the reasons could be attributed to the higher prevalence of overweight/pre-obesity in rural participants (47.55%) compared to urban participants (28.88%). Our findings contrast with a study in South Africa comparing urban and rural community-dwelling participants, which found a higher prevalence of overweight among urban older people than their rural counterparts [47]. However these comparisons should be studied with caution as an independent study conducted in an urban setting demonstrated that the BMI of older people residing in LTCFs was higher (27.5 kg/m^2^), compared to their counterparts living in the community (26.8 kg/m^2^) [44]. A systematic review conducted in Africa concurred with these results suggesting that the living environment may play a role in influencing BMI among older people [52]. The BMI patterns identified among rural-residing participants in our study mirror those frequently observed in higher-income nations, where rural urbanisation has lowered energy expenditure due to mechanisation and infrastructure advancements. Furthermore, the widespread availability of calorie-dense, nutritionally poor commercial foods has compounded the rise in BMI [53]. Countries in Sub-Saharan Africa have not followed this trend [53], however South Africa is classified as a middle-upper-income country and could account for the different BMI trend observed in rural participants from our study.

We observed a malnutrition prevalence (based on BMI < 18.5 kg/m^2^) of 2.61% in our sample. Malnutrition is associated with a higher risk of developing chronic diseases such as diabetes, cardiovascular diseases, and hypertension [54]. These conditions further complicate the health status of older people. A study conducted in Bloemfontein LTCFs found that 3.2% of older people in a higher socio-economic facility were malnourished, compared to 11.3% in a lower socio-economic institution [55]. Studies done in Sub-Saharan Africa reported malnutrition prevalence in older people ranging from 6–54% [56]. The low prevalence of malnutrition observed in our sample may be attributed to the stringent inclusion criteria and that older people in LTCFs typically experience greater food security and improved access to healthcare compared to those residing in the community [57].

Physical activity was a predictor of BMI, with increases in walking MET min/week significantly associated with decreases in BMI. A systematic review that studied the effect of walking speed and BMI on older people concurred with our findings [58]. Woo et al. [59], also found a significant impact of walking PA on BMI in older people. A study that examined the relationship between walking and BMI reported that individuals with a history of obesity had a higher likelihood of reduced walking speed and greater difficulty in maintaining mobility as they aged [60]. This suggests that maintaining a healthy BMI throughout life is crucial for preserving mobility in older people. Hip and waist circumference as well as waist-hip ratio were strongly correlated to BMI and valid predicators in the model. These associations are generally well documented in other studies conducted on older people [9, 54, 61].

### 4.5 Depression

Our analysis revealed a 35.10% prevalence of depression, as measured by the GDS, with rural participants exhibiting significantly higher levels of depression than their urban peers. A study conducted in eThekwini LTCFs reported a depression prevalence of 37.30% which aligns with our study [4]. The trend of higher depression scores in rural areas was also reported in other studies of older people in South Africa [2, 3]. Studies comparing the prevalence of depression between older people residing in LTCFs and their counterparts in community settings consistently indicate elevated depression rates among LTCFs residents [62, 63]. This disparity underscores the impact of environmental and social factors inherent in institutional living on mental health outcomes in older people. Multiple determinants may influence the elevated incidence of depression among older people in LTCFs such as social isolation/loneliness [64], loss of autonomy and independence [65], health-related issues [66], stigma and cultural factors [67, 68].

Elevated depressive states were found to predict lower BMI scores in our sample, highlighting the negative impact of depression on BMI in this cohort. These results align with a study by Ramlagan et al. [69], who reported that depression is linked to decreased food intake and weight loss in older people residing in LTCFs. Another study reported that depressive symptoms were often accompanied by weight loss and a decrease in BMI, further affecting the overall health and quality of life of residents in LTCFs [40]. A scoping review investigating the nutritional status of older people in Sub-Saharan Africa, identified depression as a significant determinant of malnutrition among residents in LTCF [56]. The review underscored that depressive symptoms, notably diminished appetite and inadequate food consumption, are major contributors to a decline in BMI within this demographic. A systematic review and meta-analysis conducted in Ethiopia, another developing country, corroborates the findings observed in our study [62].

In our analysis, BMI emerged as a significant predictor of GDS scores and may be attributed to a confluence of multifarious factors such as:

1. Inflammatory processes
  - Obesity and depression are linked to chronic low-grade inflammation, characterised by elevated pro-inflammatory cytokines such as interleukin-6 (IL-6) and C-reactive protein (CRP). Higher BMI and depressive symptoms in older people are associated with elevated levels of these variables, which can impair cognitive function and mood regulation, exacerbating depressive states [70, 71].
2. Hypothalamic-Pituitary-Adrenal (HPA) axis dysregulation
  - Dysregulation of the HPA axis, prevalent in both depression and obesity, often leads to aberrant cortisol levels that affect mood and metabolism. Chronic stress and elevated cortisol can foster fat accumulation, particularly in visceral regions, potentially intensifying depression [72, 73].
3. Serotonin and dopamine
  - Neurotransmitters, like serotonin and dopamine, are crucial for mood and appetite regulation. Serotonin deficiency is often associated with depression, while dopamine dysregulation disrupts reward pathways, influencing eating habits resulting in BMI variations [74, 75].
4. Metabolic dysfunction
  - Insulin resistance: Obesity is associated with insulin resistance and metabolic syndrome, which can impair brain function and mood. Insulin resistance may alter cerebral glucose metabolism, potentially aggravating depression [76, 77].
5. Sedentary lifestyle
  - Depression generally reduces PA, leading to caloric imbalances and BMI increases. Conversely, overweight can impair mobility and PA due to joint pain and fatigue, increasing depression and perpetuating a cycle of inactivity and weight gain [78].
6. Cultural Factors
  - Cultural norms surrounding diet, body image, and health attitudes significantly impact both weight and mental health. Societal emphasis on slimness can foster body dysmorphia and depression, while specific cultural dietary practices may influence variations in BMI [79].

The relationship between BMI and depression is best understood as a dynamic, bidirectional interaction. While higher BMI has been shown to predict increased depression risk, potentially due to physical health challenges, reduced mobility, or psychosocial factors such as stigma, higher depression scores can also lead to reductions in BMI through mechanisms such as appetite loss, poor nutrition, or metabolic dysregulation [80]. These two pathways, though unidirectional in isolation, together form a feedback loop where physical and mental health mutually influence one another over time. These findings should be approached with caution, as they stem from an analysis of cross-sectional data, which inherently restricts the capacity to ascertain precise or definitive relationships among the variables. As such, they should be regarded as indicative rather than definitive. In adolescents and middle-aged adults, the relationship between BMI and depression shares some similarities with that observed in older people, as higher BMI often predicts increased depression [81, 82]. However, a notable difference in these younger cohorts is the frequent association between increased depression and higher BMI, likely driven by behaviours such as emotional eating, reduced PA, and stress-induced metabolic changes [83–86]. The BMI-depression interplay illustrates age-specific complexities shaped by evolving physiological, psychological, and behavioural factors across the lifespan.

The findings emphasise the critical need for public health policies in South African LTCFs to implement structured PA programs and conduct routine cognitive screening. The demonstrated relationships between PA, depression, and BMI highlight the value of targeted interventions to enhance mobility, mitigate psychological disorders, and address physical health risks, ultimately improving the quality of life for older people and reducing the burden of NCDs in these facilities.

## 5 Limitations

It is imperative to acknowledge the limitations of this study to guide future research in this field. A primary limitation of this study is its cross-sectional design, which precludes the inference of causal relationships. The data provide a single temporal snapshot, capturing associations without evidence of directionality. While the findings offer valuable insights, they remain indicative rather than causative. Future longitudinal or experimental research is required to establish causality and clarify underlying mechanisms. Our rigid inclusion criteria were designed to create a homogeneous sample for robust analysis, however they inadvertently introduced selection bias. This may have impacted the generalisability of our findings by excluding less healthy participants who may exhibit different relationships between PA, BMI, and depression scores. Future studies with broader inclusion criteria are necessary to validate our findings.

A strength of our analysis was the control for confounding variables through their inclusion in regression models, minimising bias and providing more robust estimates of the relationships between variables. However, potential residual confounding from unmeasured factors, such as dietary habits or medication use, may still exist. Future research should incorporate these factors to enhance the generalisability and causal interpretation of our findings. The regression analysis effects were modest, but larger sample sizes and longitudinal analysis may yield more robust findings. Our sample was derived from one municipality in South Africa and may not accurately reflect the population of older people. A further limitation is that our rigid inclusion criteria ensured that participants were generally healthy which is also not a fair reflection of all the elderly residents in LTCFs. The outcome measures for depression and PA were self-reported and there could be inaccuracies due to memory recall challenges, particularly in older people.

## 6 Conclusion

Given the unequivocal benefits of PA in mitigating depression and managing BMI, it is crucial for LTCFs in South Africa to prioritise the implementation of structured PA regimens especially in rural residing older people. These interventions significantly bolster both physical and mental health outcomes, and by integrating such programmes, LTCFs can ensure a holistic approach to health that addresses both the physiological and psychological needs of older people, thereby promoting healthy aging.

## Supporting information

S1 Appendix Integrated care for older people screening tool. (PDF)

S2 Appendix Demographic data for sample. (PDF)

S3 Appendix Descriptive statistics and regression model outputs from SPSS V 29. (PDF)
