# Supplementary material for: Depression’s double-edged impact on body mass index. A hidden catalyst for non-communicable diseases in South Africa’s aging population in long-term care facilities
Source: PLoS One. 2025 Feb 13;20(2):e0319188. doi: 10.1371/journal.pone.0319188 (PMC11825011; doi:10.1371/journal.pone.0319188)
Supplement: S1 Appendix — (PDF) [file pone.0319188.s001.pdf]

S1 Appendix : ICOPE screening tool

WHO ICOPE SCREENING TOOL

Priority conditions associated with declines in intrinsic capacity	Tests	Assess fully any domain with a checked circle
**COGNITIVE DECLINE**	1. Remember three words: flower, door, rice (for example)	
	2. Orientation in time and space: What is the full date today? Where are you now (home, clinic, etc)?	○ Wrong to either question or does not know
	3. Recalls the three words?	○ Cannot recall all three words
**LIMITED MOBILITY**	Chair rise test: Rise from chair five times without using arms. Did the person complete five chair rises within 14 seconds?	○ No
**MALNUTRITION**	1. Weight loss: Have you unintentionally lost more than 3 kg over the last three months?	○ Yes
	2. Appetite loss: Have you experienced loss of appetite?	○ Yes
**VISUAL IMPAIRMENT**	Do you have any problems with your eyes: difficulties in seeing far, reading, eye diseases or currently under medical treatment (e.g. diabetes, high blood pressure)?	○ Yes
**HEARING LOSS**	Hears whispers (whisper test) ***or***<br>Screening audiometry result is 35 dB or less ***or***<br>Passes automated app-based digits-in-noise test	○ Fail
**DEPRESSIVE SYMPTOMS**	Over the past two weeks, have you been bothered by<br>– feeling down, depressed or hopeless?	○ Yes
	– little interest or pleasure in doing things?	○ Yes
