# Supplementary material for: Depression’s double-edged impact on body mass index. A hidden catalyst for non-communicable diseases in South Africa’s aging population in long-term care facilities
Source: PLoS One. 2025 Feb 13;20(2):e0319188. doi: 10.1371/journal.pone.0319188 (PMC11825011; doi:10.1371/journal.pone.0319188)
Supplement: S2 Appendix — (PDF) [file pone.0319188.s002.pdf]

S2 Appendix : Age and demographic data of sample

		Urban			Rural		
		Female	Male	**Total**	Female	Male	**Total**
**Age (years)**	Mean	72.54	71.65	**72.26**	77.24	72.59	**75.05**
**Marital status (*n*)**	Single	34	25	**59**	12	19	**31**
	Married	39	30	**69**	10	7	**17**
	Divorced	99	19	**118**	20	10	**30**
	Widowed	38	25	**63**	4	5	**9**
**Education level (*n*)**	No formal	4	1	**5**	5	4	**9**
	Primary school	38	7	**45**	15	10	**35**
	High school	146	76	**222**	25	25	**50**
	Diploma	14	10	**24**	1	2	**3**
	Degree	2	1	**3**	-	-	**-**

Total n = 396 : Urban female n = 210, Rural female n = 46, Urban male n = 99, Rural male n = 41
