# Supplementary material for: Depression’s double-edged impact on body mass index. A hidden catalyst for non-communicable diseases in South Africa’s aging population in long-term care facilities
Source: PLoS One. 2025 Feb 13;20(2):e0319188. doi: 10.1371/journal.pone.0319188 (PMC11825011; doi:10.1371/journal.pone.0319188)
Supplement: S3 Appendix — (PDF) [file pone.0319188.s003.pdf]

1 S3 Appendix : Descriptive statistics and regression models

1.1 Descriptive statistics

1.1.1 Stats by location and biological sex

Location 1 = Urban, 2 = Rural = Urban

Biological Sex F = 1 M = 2

Case Processing Summary							
		Cases					
		Valid		Missing		Total	
	Biological Sex F = 1 M = 2	N	Percent	N	Percent	N	Percent
Age - Years	Female	210	100.0%	0	0.0%	210	100.0%
	Male	99	100.0%	0	0.0%	99	100.0%
Resting Heart Rate (beats per minute)	Female	210	100.0%	0	0.0%	210	100.0%
	Male	99	100.0%	0	0.0%	99	100.0%
Resting Systolic Blood Pressure (mmHg)	Female	210	100.0%	0	0.0%	210	100.0%
	Male	99	100.0%	0	0.0%	99	100.0%
Resting Diastolic Blood Pressure (mmHg)	Female	210	100.0%	0	0.0%	210	100.0%
	Male	99	100.0%	0	0.0%	99	100.0%
Resting Oxygen Saturation % (SPO2)	Female	210	100.0%	0	0.0%	210	100.0%
	Male	99	100.0%	0	0.0%	99	100.0%
BMI kg/m2	Female	210	100.0%	0	0.0%	210	100.0%
	Male	99	100.0%	0	0.0%	99	100.0%
Nutritional Status - 0 = Underweight, 1 = Normal, 2 = Overweight, 3 = Obese	Female	210	100.0%	0	0.0%	210	100.0%
	Male	99	100.0%	0	0.0%	99	100.0%
Hand Grip Strength	Female	210	100.0%	0	0.0%	210	100.0%
	Male	99	100.0%	0	0.0%	99	100.0%
Hip-waist-ratio (kg)	Female	210	100.0%	0	0.0%	210	100.0%
	Male	99	100.0%	0	0.0%	99	100.0%
SPBB - Total score	Female	210	100.0%	0	0.0%	210	100.0%
	Male	99	100.0%	0	0.0%	99	100.0%
Vigrous MET/week	Female	210	100.0%	0	0.0%	210	100.0%
	Male	99	100.0%	0	0.0%	99	100.0%
Moderate MET/week	Female	210	100.0%	0	0.0%	210	100.0%
	Male	99	100.0%	0	0.0%	99	100.0%
Walking MET/week	Female	210	100.0%	0	0.0%	210	100.0%
	Male	99	100.0%	0	0.0%	99	100.0%
IPAQ - No. of minutes spent sitting per day in last 7 days	Female	210	100.0%	0	0.0%	210	100.0%
	Male	99	100.0%	0	0.0%	99	100.0%
Total MET/week	Female	210	100.0%	0	0.0%	210	100.0%
	Male	99	100.0%	0	0.0%	99	100.0%
BBAQ - Lack of time	Female	210	100.0%	0	0.0%	210	100.0%
	Male	99	100.0%	0	0.0%	99	100.0%
BBAQ - Social Influence	Female	210	100.0%	0	0.0%	210	100.0%

	Male	99	100.0%	0	0.0%	99	100.0%
BBAQ - Lack of Energy	Female	210	100.0%	0	0.0%	210	100.0%
	Male	99	100.0%	0	0.0%	99	100.0%
BBAQ - Lack of Willpower	Female	210	100.0%	0	0.0%	210	100.0%
	Male	99	100.0%	0	0.0%	99	100.0%
BBAQ - Fear of Injury	Female	210	100.0%	0	0.0%	210	100.0%
	Male	99	100.0%	0	0.0%	99	100.0%
BBAQ - Lack of Skill	Female	210	100.0%	0	0.0%	210	100.0%
	Male	99	100.0%	0	0.0%	99	100.0%
BBAQ - Lack of Resources	Female	210	100.0%	0	0.0%	210	100.0%
	Male	99	100.0%	0	0.0%	99	100.0%
GDS Total Score	Female	210	100.0%	0	0.0%	210	100.0%
	Male	99	100.0%	0	0.0%	99	100.0%
EQ-5D-3L VAS	Female	210	100.0%	0	0.0%	210	100.0%
	Male	99	100.0%	0	0.0%	99	100.0%
EQ-5D-3L Index Score	Female	210	100.0%	0	0.0%	210	100.0%
	Male	99	100.0%	0	0.0%	99	100.0%
w-FCI Total Score	Female	210	100.0%	0	0.0%	210	100.0%
	Male	99	100.0%	0	0.0%	99	100.0%
No of NCD conditions	Female	210	100.0%	0	0.0%	210	100.0%
	Male	99	100.0%	0	0.0%	99	100.0%

a. Location 1 = Urban, 2 = Rural = Urban

Descriptives[a]					
	Biological Sex F = 1 M = 2			Statistic	Std. Error
Age - Years	Female	Mean		72.54	.504
		95% Confidence Interval for Mean	Lower Bound	71.55	
			Upper Bound	73.54	
		5% Trimmed Mean		72.37	
		Median		73.00	
		Variance		53.245	
		Std. Deviation		7.297	
		Minimum		60	
		Maximum		93	
		Range		33	
		Interquartile Range		11	
		Skewness		.303	.168
		Kurtosis		-.479	.334
	Male	Mean		71.65	.767
		95% Confidence Interval for Mean	Lower Bound	70.12	
			Upper Bound	73.17	
		5% Trimmed Mean		71.33	
		Median		71.00	
		Variance		58.272	
		Std. Deviation		7.634	
		Minimum		60	
		Maximum		93	
		Range		33	
		Interquartile Range		12	
		Skewness		.498	.243
		Kurtosis		-.247	.481
Resting Heart Rate (beats per minute)	Female	Mean		78.89	.737
		95% Confidence Interval for Mean	Lower Bound	77.44	
			Upper Bound	80.34	
		5% Trimmed Mean		78.97	
		Median		79.00	
		Variance		113.954	
		Std. Deviation		10.675	
		Minimum		52	
		Maximum		100	
		Range		48	
		Interquartile Range		16	
		Skewness		-.122	.168

		Kurtosis		-.604	.334
	Male	Mean		78.07	1.150
		95% Confidence Interval for Mean	Lower Bound	75.79	
			Upper Bound	80.35	
		5% Trimmed Mean		78.14	
		Median		77.00	
		Variance		130.821	
		Std. Deviation		11.438	
		Minimum		51	
		Maximum		100	
		Range		49	
		Interquartile Range		19	
		Skewness		.021	.243
		Kurtosis		-.708	.481
Resting Systolic Blood Pressure (mmHg)	Female	Mean		130.48	.593
		95% Confidence Interval for Mean	Lower Bound	129.31	
			Upper Bound	131.65	
		5% Trimmed Mean		131.07	
		Median		132.00	
		Variance		73.887	
		Std. Deviation		8.596	
		Minimum		110	
		Maximum		140	
		Range		30	
		Interquartile Range		14	
		Skewness		-.809	.168
		Kurtosis		-.362	.334
	Male	Mean		127.71	.990
		95% Confidence Interval for Mean	Lower Bound	125.74	
			Upper Bound	129.67	
		5% Trimmed Mean		128.21	
		Median		130.00	
		Variance		97.005	
		Std. Deviation		9.849	
		Minimum		92	
		Maximum		140	
		Range		48	
		Interquartile Range		15	
		Skewness		-.713	.243
		Kurtosis		.321	.481

Resting Diastolic Blood Pressure (mmHg)	Female	Mean		76.24	.678
		95% Confidence Interval for Mean	Lower Bound	74.91	
			Upper Bound	77.58	
		5% Trimmed Mean		76.24	
		Median		76.00	
		Variance		96.434	
		Std. Deviation		9.820	
		Minimum		53	
		Maximum		100	
		Range		47	
		Interquartile Range		14	
		Skewness		-.007	.168
		Kurtosis		-.530	.334
	Male	Mean		77.05	.957
		95% Confidence Interval for Mean	Lower Bound	75.15	
			Upper Bound	78.95	
		5% Trimmed Mean		77.42	
		Median		79.00	
		Variance		90.579	
		Std. Deviation		9.517	
		Minimum		52	
		Maximum		98	
		Range		46	
		Interquartile Range		13	
		Skewness		-.492	.243
		Kurtosis		-.107	.481
Resting Oxygen Saturation % (SPO2)	Female	Mean		97.69	.069
		95% Confidence Interval for Mean	Lower Bound	97.55	
			Upper Bound	97.82	
		5% Trimmed Mean		97.72	
		Median		98.00	
		Variance		1.011	
		Std. Deviation		1.005	
		Minimum		95	
		Maximum		99	
		Range		4	
		Interquartile Range		1	
		Skewness		-.591	.168
		Kurtosis		-.516	.334
	Male	Mean		97.58	.104

		95% Confidence Interval for Mean	Lower Bound	97.37	
			Upper Bound	97.78	
		5% Trimmed Mean		97.61	
		Median		98.00	
		Variance		1.063	
		Std. Deviation		1.031	
		Minimum		95	
		Maximum		99	
		Range		4	
		Interquartile Range		1	
		Skewness		-.607	.243
		Kurtosis		-.503	.481
BMI kg/m2	Female	Mean		27.8804	.38767
		95% Confidence Interval for Mean	Lower Bound	27.1162	
			Upper Bound	28.6447	
		5% Trimmed Mean		27.7741	
		Median		27.4850	
		Variance		31.560	
		Std. Deviation		5.61785	
		Minimum		15.50	
		Maximum		41.62	
		Range		26.12	
		Interquartile Range		8.12	
		Skewness		.236	.168
		Kurtosis		-.516	.334
	Male	Mean		26.7235	.54055
		95% Confidence Interval for Mean	Lower Bound	25.6508	
			Upper Bound	27.7962	
		5% Trimmed Mean		26.5699	
		Median		25.8999	
		Variance		28.927	
		Std. Deviation		5.37840	
		Minimum		15.21	
		Maximum		39.31	
		Range		24.11	
		Interquartile Range		8.53	
		Skewness		.412	.243
		Kurtosis		-.586	.481
	Female	Mean		2.00	.060

Nutritional Status - 0 = Underweight, 1 = Normal, 2 = Overweight, 3 = Obese		95% Confidence Interval for Mean	Lower Bound	1.88	
			Upper Bound	2.12	
		5% Trimmed Mean		2.03	
		Median		2.00	
		Variance		.766	
		Std. Deviation		.875	
		Minimum		0	
		Maximum		3	
		Range		3	
		Interquartile Range		2	
		Skewness		-.216	.168
		Kurtosis		-1.195	.334
	Male	Mean		1.85	.087
		95% Confidence Interval for Mean	Lower Bound	1.68	
			Upper Bound	2.02	
		5% Trimmed Mean		1.84	
		Median		2.00	
		Variance		.742	
		Std. Deviation		.861	
		Minimum		0	
		Maximum		3	
		Range		3	
		Interquartile Range		2	
		Skewness		.202	.243
		Kurtosis		-1.431	.481
Hand Grip Strength	Female	Mean		14.4562	.38675
		95% Confidence Interval for Mean	Lower Bound	13.6938	
			Upper Bound	15.2186	
		5% Trimmed Mean		14.1847	
		Median		14.1000	
		Variance		31.411	
		Std. Deviation		5.60456	
		Minimum		3.40	
		Maximum		38.30	
		Range		34.90	
		Interquartile Range		8.02	
		Skewness		.833	.168
		Kurtosis		1.457	.334
	Male	Mean		19.9010	.96451

		95% Confidence Interval for Mean	Lower Bound	17.9870	
			Upper Bound	21.8151	
		5% Trimmed Mean		19.3499	
		Median		18.0000	
		Variance		92.098	
		Std. Deviation		9.59676	
		Minimum		4.50	
		Maximum		45.90	
		Range		41.40	
		Interquartile Range		14.90	
		Skewness		.746	.243
		Kurtosis		-.230	.481
Hip-waist-ratio (kg)	Female	Mean		.9069	.00670
		95% Confidence Interval for Mean	Lower Bound	.8937	
			Upper Bound	.9201	
		5% Trimmed Mean		.9000	
		Median		.8935	
		Variance		.009	
		Std. Deviation		.09705	
		Minimum		.71	
		Maximum		1.59	
		Range		.88	
		Interquartile Range		.09	
		Skewness		2.569	.168
		Kurtosis		13.766	.334
	Male	Mean		.9922	.01615
		95% Confidence Interval for Mean	Lower Bound	.9601	
			Upper Bound	1.0242	
		5% Trimmed Mean		.9793	
		Median		.9592	
		Variance		.026	
		Std. Deviation		.16068	
		Minimum		.64	
		Maximum		2.15	
		Range		1.50	
		Interquartile Range		.17	
		Skewness		3.978	.243
		Kurtosis		26.922	.481
SPBB - Total score	Female	Mean		7.01	.226

		95% Confidence Interval for Mean	Lower Bound	6.56	
			Upper Bound	7.46	
		5% Trimmed Mean		7.09	
		Median		7.00	
		Variance		10.756	
		Std. Deviation		3.280	
		Minimum		0	
		Maximum		12	
		Range		12	
		Interquartile Range		5	
		Skewness		-.308	.168
		Kurtosis		-.866	.334
	Male	Mean		6.53	.338
		95% Confidence Interval for Mean	Lower Bound	5.85	
			Upper Bound	7.20	
		5% Trimmed Mean		6.56	
		Median		7.00	
		Variance		11.334	
		Std. Deviation		3.367	
		Minimum		0	
		Maximum		12	
		Range		12	
		Interquartile Range		5	
		Skewness		-.156	.243
		Kurtosis		-.932	.481
Vigrous MET/week	Female	Mean		209.3333	66.02196
		95% Confidence Interval for Mean	Lower Bound	79.1790	
			Upper Bound	339.4877	
		5% Trimmed Mean		35.0794	
		Median		.0000	
		Variance		915368.931	
		Std. Deviation		956.74915	
		Minimum		.00	
		Maximum		10080.00	
		Range		10080.00	
		Interquartile Range		.00	
		Skewness		7.018	.168
		Kurtosis		60.635	.334
	Male	Mean		124.6465	64.91377

		95% Confidence Interval for Mean	Lower Bound	-4.1728	
			Upper Bound	253.4657	
		5% Trimmed Mean		3.3109	
		Median		.0000	
		Variance		417165.945	
		Std. Deviation		645.88385	
		Minimum		.00	
		Maximum		4480.00	
		Range		4480.00	
		Interquartile Range		.00	
		Skewness		6.148	.243
		Kurtosis		38.819	.481
Moderate MET/week	Female	Mean		476.1905	53.80423
		95% Confidence Interval for Mean	Lower Bound	370.1219	
			Upper Bound	582.2590	
		5% Trimmed Mean		355.7672	
		Median		140.0000	
		Variance		607928.002	
		Std. Deviation		779.69738	
		Minimum		.00	
		Maximum		4320.00	
		Range		4320.00	
		Interquartile Range		720.00	
		Skewness		2.611	.168
		Kurtosis		7.773	.334
	Male	Mean		422.0606	78.35376
		95% Confidence Interval for Mean	Lower Bound	266.5701	
			Upper Bound	577.5511	
		5% Trimmed Mean		300.2020	
		Median		120.0000	
		Variance		607791.792	
		Std. Deviation		779.61003	
		Minimum		.00	
		Maximum		5040.00	
		Range		5040.00	
		Interquartile Range		560.00	
		Skewness		3.702	.243
		Kurtosis		17.213	.481
Walking MET/week	Female	Mean		1094.5786	79.07151

		95% Confidence Interval for Mean	Lower Bound	938.6986	
			Upper Bound	1250.4585	
		5% Trimmed Mean		985.1984	
		Median		643.5000	
		Variance		1312983.794	
		Std. Deviation		1145.85505	
		Minimum		.00	
		Maximum		4158.00	
		Range		4158.00	
		Interquartile Range		1138.50	
		Skewness		1.320	.168
		Kurtosis		.908	.334
	Male	Mean		1214.6667	118.79669
		95% Confidence Interval for Mean	Lower Bound	978.9185	
			Upper Bound	1450.4148	
		5% Trimmed Mean		1118.6296	
		Median		792.0000	
		Variance		1397152.702	
		Std. Deviation		1182.01214	
		Minimum		.00	
		Maximum		4158.00	
		Range		4158.00	
		Interquartile Range		1732.50	
		Skewness		1.282	.243
		Kurtosis		.745	.481
IPAQ - No. of minutes spent sitting per day in last 7 days	Female	Mean		151.64	3.088
		95% Confidence Interval for Mean	Lower Bound	145.55	
			Upper Bound	157.73	
		5% Trimmed Mean		156.61	
		Median		180.00	
		Variance		2002.671	
		Std. Deviation		44.751	
		Minimum		15	
		Maximum		180	
		Range		165	
		Interquartile Range		60	
		Skewness		-1.433	.168
		Kurtosis		1.040	.334
	Male	Mean		153.54	4.767

		95% Confidence Interval for Mean	Lower Bound	144.08	
			Upper Bound	163.00	
		5% Trimmed Mean		159.38	
		Median		180.00	
		Variance		2249.619	
		Std. Deviation		47.430	
		Minimum		10	
		Maximum		180	
		Range		170	
		Interquartile Range		60	
		Skewness		-1.682	.243
		Kurtosis		1.599	.481
Total MET/week	Female	Mean		1693.9524	104.19550
		95% Confidence Interval for Mean	Lower Bound	1488.5435	
			Upper Bound	1899.3613	
		5% Trimmed Mean		1593.0704	
		Median		1201.5000	
		Variance		2279907.548	
		Std. Deviation		1509.93627	
		Minimum		66.00	
		Maximum		5172.00	
		Range		5106.00	
		Interquartile Range		2370.00	
		Skewness		.915	.168
		Kurtosis		-.496	.334
	Male	Mean		1674.7273	139.69454
		95% Confidence Interval for Mean	Lower Bound	1397.5080	
			Upper Bound	1951.9466	
		5% Trimmed Mean		1584.6431	
		Median		1228.5000	
		Variance		1931942.021	
		Std. Deviation		1389.94317	
		Minimum		99.00	
		Maximum		5172.00	
		Range		5073.00	
		Interquartile Range		1849.30	
		Skewness		.989	.243
		Kurtosis		-.174	.481
BBAQ - Lack of time	Female	Mean		2.71	.183

		95% Confidence Interval for Mean	Lower Bound	2.35	
			Upper Bound	3.08	
		5% Trimmed Mean		2.53	
		Median		2.00	
		Variance		7.066	
		Std. Deviation		2.658	
		Minimum		0	
		Maximum		9	
		Range		9	
		Interquartile Range		5	
		Skewness		.691	.168
		Kurtosis		-.580	.334
	Male	Mean		3.08	.281
		95% Confidence Interval for Mean	Lower Bound	2.52	
			Upper Bound	3.64	
		5% Trimmed Mean		2.92	
		Median		2.00	
		Variance		7.830	
		Std. Deviation		2.798	
		Minimum		0	
		Maximum		9	
		Range		9	
		Interquartile Range		6	
		Skewness		.608	.243
		Kurtosis		-.678	.481
BBAQ - Social Influence	Female	Mean		2.85	.187
		95% Confidence Interval for Mean	Lower Bound	2.48	
			Upper Bound	3.22	
		5% Trimmed Mean		2.67	
		Median		3.00	
		Variance		7.316	
		Std. Deviation		2.705	
		Minimum		0	
		Maximum		9	
		Range		9	
		Interquartile Range		5	
		Skewness		.645	.168
		Kurtosis		-.611	.334
	Male	Mean		3.47	.283

		95% Confidence Interval for Mean	Lower Bound	2.91	
			Upper Bound	4.04	
		5% Trimmed Mean		3.36	
		Median		3.00	
		Variance		7.946	
		Std. Deviation		2.819	
		Minimum		0	
		Maximum		9	
		Range		9	
		Interquartile Range		5	
		Skewness		.645	.243
		Kurtosis		-.564	.481
BBAQ - Lack of Energy	Female	Mean		2.61	.179
		95% Confidence Interval for Mean	Lower Bound	2.26	
			Upper Bound	2.97	
		5% Trimmed Mean		2.41	
		Median		2.00	
		Variance		6.745	
		Std. Deviation		2.597	
		Minimum		0	
		Maximum		9	
		Range		9	
		Interquartile Range		4	
		Skewness		.821	.168
		Kurtosis		-.218	.334
	Male	Mean		3.14	.259
		95% Confidence Interval for Mean	Lower Bound	2.63	
			Upper Bound	3.66	
		5% Trimmed Mean		2.99	
		Median		3.00	
		Variance		6.653	
		Std. Deviation		2.579	
		Minimum		0	
		Maximum		9	
		Range		9	
		Interquartile Range		4	
		Skewness		.638	.243
		Kurtosis		-.320	.481
BBAQ - Lack of Willpower	Female	Mean		3.44	.189

		95% Confidence Interval for Mean	Lower Bound	3.06	
			Upper Bound	3.81	
		5% Trimmed Mean		3.32	
		Median		3.00	
		Variance		7.530	
		Std. Deviation		2.744	
		Minimum		0	
		Maximum		9	
		Range		9	
		Interquartile Range		4	
		Skewness		.437	.168
		Kurtosis		-.816	.334
	Male	Mean		4.36	.446
		95% Confidence Interval for Mean	Lower Bound	3.48	
			Upper Bound	5.25	
		5% Trimmed Mean		4.01	
		Median		3.00	
		Variance		19.683	
		Std. Deviation		4.437	
		Minimum		0	
		Maximum		39	
		Range		39	
		Interquartile Range		4	
		Skewness		4.905	.243
		Kurtosis		37.494	.481
BBAQ - Fear of Injury	Female	Mean		2.81	.184
		95% Confidence Interval for Mean	Lower Bound	2.45	
			Upper Bound	3.17	
		5% Trimmed Mean		2.63	
		Median		2.00	
		Variance		7.141	
		Std. Deviation		2.672	
		Minimum		0	
		Maximum		9	
		Range		9	
		Interquartile Range		5	
		Skewness		.698	.168
		Kurtosis		-.535	.334
	Male	Mean		3.21	.261

		95% Confidence Interval for Mean	Lower Bound	2.69	
			Upper Bound	3.73	
		5% Trimmed Mean		3.08	
		Median		3.00	
		Variance		6.761	
		Std. Deviation		2.600	
		Minimum		0	
		Maximum		9	
		Range		9	
		Interquartile Range		4	
		Skewness		.441	.243
		Kurtosis		-.676	.481
BBAQ - Lack of Skill	Female	Mean		2.91	.181
		95% Confidence Interval for Mean	Lower Bound	2.55	
			Upper Bound	3.27	
		5% Trimmed Mean		2.74	
		Median		3.00	
		Variance		6.858	
		Std. Deviation		2.619	
		Minimum		0	
		Maximum		9	
		Range		9	
		Interquartile Range		4	
		Skewness		.726	.168
		Kurtosis		-.384	.334
	Male	Mean		3.33	.273
		95% Confidence Interval for Mean	Lower Bound	2.79	
			Upper Bound	3.87	
		5% Trimmed Mean		3.20	
		Median		3.00	
		Variance		7.367	
		Std. Deviation		2.714	
		Minimum		0	
		Maximum		9	
		Range		9	
		Interquartile Range		4	
		Skewness		.580	.243
		Kurtosis		-.561	.481
BBAQ - Lack of Resources	Female	Mean		4.60	.208

		95% Confidence Interval for Mean	Lower Bound	4.19	
			Upper Bound	5.00	
		5% Trimmed Mean		4.61	
		Median		5.00	
		Variance		9.046	
		Std. Deviation		3.008	
		Minimum		0	
		Maximum		9	
		Range		9	
		Interquartile Range		5	
		Skewness		-.039	.168
		Kurtosis		-1.223	.334
	Male	Mean		5.05	.268
		95% Confidence Interval for Mean	Lower Bound	4.52	
			Upper Bound	5.58	
		5% Trimmed Mean		5.11	
		Median		6.00	
		Variance		7.089	
		Std. Deviation		2.663	
		Minimum		0	
		Maximum		9	
		Range		9	
		Interquartile Range		4	
		Skewness		-.218	.243
		Kurtosis		-.875	.481
GDS Total Score	Female	Mean		3.56	.209
		95% Confidence Interval for Mean	Lower Bound	3.15	
			Upper Bound	3.97	
		5% Trimmed Mean		3.33	
		Median		3.00	
		Variance		9.195	
		Std. Deviation		3.032	
		Minimum		0	
		Maximum		14	
		Range		14	
		Interquartile Range		4	
		Skewness		1.031	.168
		Kurtosis		.483	.334
	Male	Mean		4.02	.297

		95% Confidence Interval for Mean	Lower Bound	3.43	
			Upper Bound	4.61	
		5% Trimmed Mean		3.82	
		Median		3.00	
		Variance		8.734	
		Std. Deviation		2.955	
		Minimum		0	
		Maximum		13	
		Range		13	
		Interquartile Range		3	
		Skewness		1.004	.243
		Kurtosis		.407	.481
EQ-5D-3L VAS	Female	Mean		70.30	1.161
		95% Confidence Interval for Mean	Lower Bound	68.01	
			Upper Bound	72.58	
		5% Trimmed Mean		70.65	
		Median		70.00	
		Variance		282.850	
		Std. Deviation		16.818	
		Minimum		20	
		Maximum		100	
		Range		80	
		Interquartile Range		20	
		Skewness		-.331	.168
		Kurtosis		-.353	.334
	Male	Mean		71.52	1.736
		95% Confidence Interval for Mean	Lower Bound	68.07	
			Upper Bound	74.96	
		5% Trimmed Mean		71.91	
		Median		70.00	
		Variance		298.191	
		Std. Deviation		17.268	
		Minimum		20	
		Maximum		100	
		Range		80	
		Interquartile Range		25	
		Skewness		-.300	.243
		Kurtosis		-.214	.481
EQ-5D-3L Index Score	Female	Mean		.8161	.01040

		95% Confidence Interval for Mean	Lower Bound	.7956	
			Upper Bound	.8366	
		5% Trimmed Mean		.8263	
		Median		.8330	
		Variance		.023	
		Std. Deviation		.15078	
		Minimum		.27	
		Maximum		1.00	
		Range		.73	
		Interquartile Range		.25	
		Skewness		-.750	.168
		Kurtosis		.637	.334
	Male	Mean		.8503	.01185
		95% Confidence Interval for Mean	Lower Bound	.8268	
			Upper Bound	.8738	
		5% Trimmed Mean		.8571	
		Median		.8330	
		Variance		.014	
		Std. Deviation		.11793	
		Minimum		.38	
		Maximum		1.00	
		Range		.62	
		Interquartile Range		.21	
		Skewness		-.569	.243
		Kurtosis		1.404	.481
w-FCI Total Score	Female	Mean		4.68	.244
		95% Confidence Interval for Mean	Lower Bound	4.20	
			Upper Bound	5.16	
		5% Trimmed Mean		4.44	
		Median		4.00	
		Variance		12.486	
		Std. Deviation		3.534	
		Minimum		0	
		Maximum		19	
		Range		19	
		Interquartile Range		5	
		Skewness		.996	.168
		Kurtosis		1.330	.334
	Male	Mean		3.91	.284

		95% Confidence Interval for Mean	Lower Bound	3.35	
			Upper Bound	4.47	
		5% Trimmed Mean		3.71	
		Median		3.00	
		Variance		7.981	
		Std. Deviation		2.825	
		Minimum		0	
		Maximum		17	
		Range		17	
		Interquartile Range		4	
		Skewness		1.377	.243
		Kurtosis		3.808	.481
No of NCD conditions	Female	Mean		3.14	.144
		95% Confidence Interval for Mean	Lower Bound	2.85	
			Upper Bound	3.42	
		5% Trimmed Mean		3.05	
		Median		3.00	
		Variance		4.349	
		Std. Deviation		2.085	
		Minimum		0	
		Maximum		10	
		Range		10	
		Interquartile Range		2	
		Skewness		.575	.168
		Kurtosis		-.058	.334
	Male	Mean		2.96	.187
		95% Confidence Interval for Mean	Lower Bound	2.59	
			Upper Bound	3.33	
		5% Trimmed Mean		2.87	
		Median		3.00	
		Variance		3.468	
		Std. Deviation		1.862	
		Minimum		0	
		Maximum		9	
		Range		9	
		Interquartile Range		2	
		Skewness		.815	.243
		Kurtosis		.639	.481

a. Location 1 = Urban, 2 = Rural = Urban

Location 1 = Urban, 2 = Rural = Rural

Biological Sex F = 1 M = 2

Case Processing Summary[a]

		Cases					
		Valid		Missing		Total	
	Biological Sex F = 1 M = 2	N	Percent	N	Percent	N	Percent
Age - Years	Female	46	100.0%	0	0.0%	46	100.0%
	Male	41	100.0%	0	0.0%	41	100.0%
Resting Heart Rate (beats per minute)	Female	46	100.0%	0	0.0%	46	100.0%
	Male	41	100.0%	0	0.0%	41	100.0%
Resting Systolic Blood Pressure (mmHg)	Female	46	100.0%	0	0.0%	46	100.0%
	Male	41	100.0%	0	0.0%	41	100.0%
Resting Diastolic Blood Pressure (mmHg)	Female	46	100.0%	0	0.0%	46	100.0%
	Male	41	100.0%	0	0.0%	41	100.0%
Resting Oxygen Saturation % (SPO2)	Female	46	100.0%	0	0.0%	46	100.0%
	Male	41	100.0%	0	0.0%	41	100.0%
BMI kg/m2	Female	46	100.0%	0	0.0%	46	100.0%
	Male	41	100.0%	0	0.0%	41	100.0%
Nutritional Status - 0 = Underweight, 1 = Normal, 2 = Overweight, 3 = Obese	Female	46	100.0%	0	0.0%	46	100.0%
	Male	41	100.0%	0	0.0%	41	100.0%
Hand Grip Strength	Female	46	100.0%	0	0.0%	46	100.0%
	Male	41	100.0%	0	0.0%	41	100.0%
Hip-waist-ratio (kg)	Female	46	100.0%	0	0.0%	46	100.0%
	Male	41	100.0%	0	0.0%	41	100.0%
SPBB - Total score	Female	46	100.0%	0	0.0%	46	100.0%
	Male	41	100.0%	0	0.0%	41	100.0%
Vigrous MET/week	Female	46	100.0%	0	0.0%	46	100.0%
	Male	41	100.0%	0	0.0%	41	100.0%
Moderate MET/week	Female	46	100.0%	0	0.0%	46	100.0%
	Male	41	100.0%	0	0.0%	41	100.0%
Walking MET/week	Female	46	100.0%	0	0.0%	46	100.0%
	Male	41	100.0%	0	0.0%	41	100.0%
IPAQ - No. of minutes spent sitting per day in last 7 days	Female	46	100.0%	0	0.0%	46	100.0%
	Male	41	100.0%	0	0.0%	41	100.0%
Total MET/week	Female	46	100.0%	0	0.0%	46	100.0%
	Male	41	100.0%	0	0.0%	41	100.0%
BBAQ - Lack of time	Female	46	100.0%	0	0.0%	46	100.0%
	Male	41	100.0%	0	0.0%	41	100.0%
BBAQ - Social Influence	Female	46	100.0%	0	0.0%	46	100.0%
	Male	41	100.0%	0	0.0%	41	100.0%
BBAQ - Lack of Energy	Female	46	100.0%	0	0.0%	46	100.0%
	Male	41	100.0%	0	0.0%	41	100.0%
BBAQ - Lack of Willpower	Female	46	100.0%	0	0.0%	46	100.0%
	Male	41	100.0%	0	0.0%	41	100.0%
BBAQ - Fear of Injury	Female	46	100.0%	0	0.0%	46	100.0%
	Male	41	100.0%	0	0.0%	41	100.0%
BBAQ - Lack of Skill	Female	46	100.0%	0	0.0%	46	100.0%

	Male	41	100.0%	0	0.0%	41	100.0%
BBAQ - Lack of Resources	Female	46	100.0%	0	0.0%	46	100.0%
	Male	41	100.0%	0	0.0%	41	100.0%
GDS Total Score	Female	46	100.0%	0	0.0%	46	100.0%
	Male	41	100.0%	0	0.0%	41	100.0%
EQ-5D-3L VAS	Female	46	100.0%	0	0.0%	46	100.0%
	Male	41	100.0%	0	0.0%	41	100.0%
EQ-5D-3L Index Score	Female	46	100.0%	0	0.0%	46	100.0%
	Male	41	100.0%	0	0.0%	41	100.0%
w-FCI Total Score	Female	46	100.0%	0	0.0%	46	100.0%
	Male	41	100.0%	0	0.0%	41	100.0%
No of NCD conditions	Female	46	100.0%	0	0.0%	46	100.0%
	Male	41	100.0%	0	0.0%	41	100.0%

a. Location 1 = Urban, 2 = Rural = Rural

Descriptives[a]

	Biological Sex F = 1 M = 2			Statistic	Std. Error
Age - Years	Female	Mean		77.24	1.063
		95% Confidence Interval for Mean	Lower Bound	75.10	
			Upper Bound	79.38	
		5% Trimmed Mean		77.01	
		Median		76.50	
		Variance		52.008	
		Std. Deviation		7.212	
		Minimum		64	
		Maximum		98	
		Range		34	
		Interquartile Range		10	
		Skewness		.560	.350
		Kurtosis		.323	.688
	Male	Mean		72.59	1.397
		95% Confidence Interval for Mean	Lower Bound	69.76	
			Upper Bound	75.41	
		5% Trimmed Mean		72.27	
		Median		72.00	
		Variance		79.999	
		Std. Deviation		8.944	
		Minimum		56	
		Maximum		93	
		Range		37	
		Interquartile Range		14	
		Skewness		.521	.369
		Kurtosis		-.166	.724
Resting Heart Rate (beats per minute)	Female	Mean		81.04	1.400
		95% Confidence Interval for Mean	Lower Bound	78.22	

			Upper Bound	83.86	
		5% Trimmed Mean		81.48	
		Median		82.00	
		Variance		90.176	
		Std. Deviation		9.496	
		Minimum		57	
		Maximum		98	
		Range		41	
		Interquartile Range		10	
		Skewness		-.872	.350
		Kurtosis		.908	.688
	Male	Mean		78.41	1.780
		95% Confidence Interval for Mean	Lower Bound	74.82	
			Upper Bound	82.01	
		5% Trimmed Mean		78.62	
		Median		78.00	
		Variance		129.899	
		Std. Deviation		11.397	
		Minimum		51	
		Maximum		100	
		Range		49	
		Interquartile Range		18	
		Skewness		-.175	.369
		Kurtosis		-.383	.724
Resting Systolic Blood Pressure (mmHg)	Female	Mean		132.61	1.104
		95% Confidence Interval for Mean	Lower Bound	130.39	
			Upper Bound	134.83	
		5% Trimmed Mean		133.32	
		Median		133.00	
		Variance		56.021	
		Std. Deviation		7.485	
		Minimum		107	
		Maximum		140	
		Range		33	
		Interquartile Range		10	
		Skewness		-1.394	.350
		Kurtosis		2.089	.688
	Male	Mean		126.32	1.733
		95% Confidence Interval for Mean	Lower Bound	122.81	
			Upper Bound	129.82	

		5% Trimmed Mean		127.01	
		Median		128.00	
		Variance		123.172	
		Std. Deviation		11.098	
		Minimum		100	
		Maximum		140	
		Range		40	
		Interquartile Range		17	
		Skewness		-.764	.369
		Kurtosis		-.219	.724
Resting Diastolic Blood Pressure (mmHg)	Female	Mean		80.98	1.153
		95% Confidence Interval for Mean	Lower Bound	78.66	
			Upper Bound	83.30	
		5% Trimmed Mean		81.37	
		Median		80.50	
		Variance		61.133	
		Std. Deviation		7.819	
		Minimum		59	
		Maximum		92	
		Range		33	
		Interquartile Range		12	
		Skewness		-.636	.350
		Kurtosis		.084	.688
	Male	Mean		77.80	1.348
		95% Confidence Interval for Mean	Lower Bound	75.08	
			Upper Bound	80.53	
		5% Trimmed Mean		78.19	
		Median		78.00	
		Variance		74.461	
		Std. Deviation		8.629	
		Minimum		52	
		Maximum		93	
		Range		41	
		Interquartile Range		11	
		Skewness		-.617	.369
		Kurtosis		.854	.724
Resting Oxygen Saturation % (SPO2)	Female	Mean		98.00	.132
		95% Confidence Interval for Mean	Lower Bound	97.73	
			Upper Bound	98.27	
		5% Trimmed Mean		98.06	
		Median		98.00	

				Statistic	Std. Error
		Variance		.800	
		Std. Deviation		.894	
		Minimum		96	
		Maximum		99	
		Range		3	
		Interquartile Range		1	
		Skewness		-.974	.350
		Kurtosis		.598	.688
	Male	Mean		97.98	.150
		95% Confidence Interval for Mean	Lower Bound	97.67	
			Upper Bound	98.28	
		5% Trimmed Mean		98.03	
		Median		98.00	
		Variance		.924	
		Std. Deviation		.961	
		Minimum		96	
		Maximum		99	
		Range		3	
		Interquartile Range		1	
		Skewness		-.837	.369
		Kurtosis		-.040	.724
BMI kg/m2	Female	Mean		27.7683	.87000
		95% Confidence Interval for Mean	Lower Bound	26.0161	
			Upper Bound	29.5206	
		5% Trimmed Mean		27.6402	
		Median		25.9504	
		Variance		34.818	
		Std. Deviation		5.90065	
		Minimum		16.19	
		Maximum		40.18	
		Range		23.99	
		Interquartile Range		8.08	
		Skewness		.652	.350
		Kurtosis		-.152	.688
	Male	Mean		27.4036	.70556
		95% Confidence Interval for Mean	Lower Bound	25.9776	
			Upper Bound	28.8296	
		5% Trimmed Mean		27.3038	
		Median		26.0790	
		Variance		20.410	
		Std. Deviation		4.51779	

		Minimum		16.87	
		Maximum		38.97	
		Range		22.11	
		Interquartile Range		5.50	
		Skewness		.426	.369
		Kurtosis		.745	.724
Nutritional Status - 0 = Underweight, 1 = Normal, 2 = Overweight, 3 = Obese	Female	Mean		1.80	.130
		95% Confidence Interval for Mean	Lower Bound	1.54	
			Upper Bound	2.07	
		5% Trimmed Mean		1.83	
		Median		2.00	
		Variance		.783	
		Std. Deviation		.885	
		Minimum		0	
		Maximum		3	
		Range		3	
		Interquartile Range		2	
		Skewness		.001	.350
		Kurtosis		-1.022	.688
	Male	Mean		1.93	.123
		95% Confidence Interval for Mean	Lower Bound	1.68	
			Upper Bound	2.18	
		5% Trimmed Mean		1.95	
		Median		2.00	
		Variance		.620	
		Std. Deviation		.787	
		Minimum		0	
		Maximum		3	
		Range		3	
		Interquartile Range		2	
		Skewness		-.191	.369
		Kurtosis		-.577	.724
Hand Grip Strength	Female	Mean		12.8500	.67657
		95% Confidence Interval for Mean	Lower Bound	11.4873	
			Upper Bound	14.2127	
		5% Trimmed Mean		12.5857	
		Median		12.6000	
		Variance		21.056	
		Std. Deviation		4.58872	
		Minimum		2.40	
		Maximum		31.00	

		Range		28.60	
		Interquartile Range		4.73	
		Skewness		1.341	.350
		Kurtosis		4.794	.688
	Male	Mean		24.1854	1.49268
		95% Confidence Interval for Mean	Lower Bound	21.1686	
			Upper Bound	27.2022	
		5% Trimmed Mean		23.8344	
		Median		25.8000	
		Variance		91.351	
		Std. Deviation		9.55779	
		Minimum		8.30	
		Maximum		45.70	
		Range		37.40	
		Interquartile Range		14.65	
		Skewness		.201	.369
		Kurtosis		-.444	.724
Hip-waist-ratio (kg)	Female	Mean		.9092	.01272
		95% Confidence Interval for Mean	Lower Bound	.8836	
			Upper Bound	.9349	
		5% Trimmed Mean		.9064	
		Median		.8992	
		Variance		.007	
		Std. Deviation		.08629	
		Minimum		.71	
		Maximum		1.17	
		Range		.46	
		Interquartile Range		.09	
		Skewness		.610	.350
		Kurtosis		1.618	.688
	Male	Mean		.9424	.02217
		95% Confidence Interval for Mean	Lower Bound	.8976	
			Upper Bound	.9872	
		5% Trimmed Mean		.9234	
		Median		.8992	
		Variance		.020	
		Std. Deviation		.14193	
		Minimum		.81	
		Maximum		1.64	
		Range		.83	
		Interquartile Range		.11	

		Skewness		3.225	.369
		Kurtosis		14.191	.724
SPBB - Total score	Female	Mean		5.85	.416
		95% Confidence Interval for Mean	Lower Bound	5.01	
			Upper Bound	6.69	
		5% Trimmed Mean		5.81	
		Median		6.00	
		Variance		7.954	
		Std. Deviation		2.820	
		Minimum		1	
		Maximum		12	
		Range		11	
		Interquartile Range		4	
		Skewness		.057	.350
		Kurtosis		-.420	.688
	Male	Mean		6.68	.428
		95% Confidence Interval for Mean	Lower Bound	5.82	
			Upper Bound	7.55	
		5% Trimmed Mean		6.73	
		Median		7.00	
		Variance		7.522	
		Std. Deviation		2.743	
		Minimum		1	
		Maximum		12	
		Range		11	
		Interquartile Range		4	
		Skewness		-.422	.369
		Kurtosis		-.440	.724
Vigrous MET/week	Female	Mean		126.0870	64.31766
		95% Confidence Interval for Mean	Lower Bound	-3.4555	
			Upper Bound	255.6294	
		5% Trimmed Mean		40.5797	
		Median		.0000	
		Variance		190291.014	
		Std. Deviation		436.22358	
		Minimum		.00	
		Maximum		2080.00	
		Range		2080.00	
		Interquartile Range		.00	
		Skewness		3.563	.350
		Kurtosis		12.222	.688
	Male	Mean		275.1220	245.72122

		95% Confidence Interval for Mean	Lower Bound	-221.4992	
			Upper Bound	771.7431	
		5% Trimmed Mean		18.8618	
		Median		.0000	
		Variance		2475535.610	
		Std. Deviation		1573.38349	
		Minimum		.00	
		Maximum		10080.00	
		Range		10080.00	
		Interquartile Range		.00	
		Skewness		6.355	.369
		Kurtosis		40.570	.724
Moderate MET/week	Female	Mean		292.6087	101.29663
		95% Confidence Interval for Mean	Lower Bound	88.5868	
			Upper Bound	496.6306	
		5% Trimmed Mean		178.7440	
		Median		.0000	
		Variance		472006.377	
		Std. Deviation		687.02720	
		Minimum		.00	
		Maximum		3360.00	
		Range		3360.00	
		Interquartile Range		150.00	
		Skewness		2.983	.350
		Kurtosis		9.425	.688
	Male	Mean		222.9268	85.10413
		95% Confidence Interval for Mean	Lower Bound	50.9250	
			Upper Bound	394.9287	
		5% Trimmed Mean		132.8997	
		Median		.0000	
		Variance		296951.220	
		Std. Deviation		544.93231	
		Minimum		.00	
		Maximum		3360.00	
		Range		3360.00	
		Interquartile Range		340.00	
		Skewness		5.034	.369
		Kurtosis		28.756	.724
Walking MET/week	Female	Mean		1007.9348	164.82281
		95% Confidence Interval for Mean	Lower Bound	675.9646	

			Upper Bound	1339.9050	
		5% Trimmed Mean		888.9275	
		Median		643.5000	
		Variance		1249661.696	
		Std. Deviation		1117.88268	
		Minimum		.00	
		Maximum		4158.00	
		Range		4158.00	
		Interquartile Range		1155.00	
		Skewness		1.946	.350
		Kurtosis		3.362	.688
	Male	Mean		991.6098	132.94462
		95% Confidence Interval for Mean	Lower Bound	722.9187	
			Upper Bound	1260.3009	
		5% Trimmed Mean		910.0488	
		Median		693.0000	
		Variance		724645.169	
		Std. Deviation		851.26093	
		Minimum		.00	
		Maximum		4158.00	
		Range		4158.00	
		Interquartile Range		924.00	
		Skewness		1.857	.369
		Kurtosis		4.254	.724
IPAQ - No. of minutes spent sitting per day in last 7 days	Female	Mean		150.43	7.216
		95% Confidence Interval for Mean	Lower Bound	135.90	
			Upper Bound	164.97	
		5% Trimmed Mean		156.04	
		Median		180.00	
		Variance		2395.362	
		Std. Deviation		48.942	
		Minimum		20	
		Maximum		180	
		Range		160	
		Interquartile Range		60	
		Skewness		-1.573	.350
		Kurtosis		1.472	.688
	Male	Mean		157.56	6.706
		95% Confidence Interval for Mean	Lower Bound	144.01	
			Upper Bound	171.11	

		5% Trimmed Mean		163.90	
		Median		180.00	
		Variance		1843.902	
		Std. Deviation		42.941	
		Minimum		20	
		Maximum		180	
		Range		160	
		Interquartile Range		60	
		Skewness		-2.065	.369
		Kurtosis		3.855	.724
Total MET/week	Female	Mean		1401.7174	205.13471
		95% Confidence Interval for Mean	Lower Bound	988.5549	
			Upper Bound	1814.8799	
		5% Trimmed Mean		1291.3575	
		Median		939.0000	
		Variance		1935691.485	
		Std. Deviation		1391.29130	
		Minimum		20.00	
		Maximum		5172.00	
		Range		5152.00	
		Interquartile Range		1315.88	
		Skewness		1.338	.350
		Kurtosis		.644	.688
	Male	Mean		1267.2195	196.14193
		95% Confidence Interval for Mean	Lower Bound	870.8019	
			Upper Bound	1663.6371	
		5% Trimmed Mean		1115.5738	
		Median		1039.5000	
		Variance		1577337.901	
		Std. Deviation		1255.92114	
		Minimum		60.00	
		Maximum		5172.00	
		Range		5112.00	
		Interquartile Range		870.00	
		Skewness		2.252	.369
		Kurtosis		4.775	.724
BBAQ - Lack of time	Female	Mean		2.61	.322
		95% Confidence Interval for Mean	Lower Bound	1.96	
			Upper Bound	3.26	
		5% Trimmed Mean		2.51	
		Median		2.00	

		Variance		4.777	
		Std. Deviation		2.186	
		Minimum		0	
		Maximum		7	
		Range		7	
		Interquartile Range		3	
		Skewness		.474	.350
		Kurtosis		-.864	.688
	Male	Mean		2.51	.412
		95% Confidence Interval for Mean	Lower Bound	1.68	
			Upper Bound	3.34	
		5% Trimmed Mean		2.32	
		Median		1.00	
		Variance		6.956	
		Std. Deviation		2.637	
		Minimum		0	
		Maximum		9	
		Range		9	
		Interquartile Range		5	
		Skewness		.948	.369
		Kurtosis		-.290	.724
BBAQ - Social Influence	Female	Mean		2.87	.301
		95% Confidence Interval for Mean	Lower Bound	2.26	
			Upper Bound	3.48	
		5% Trimmed Mean		2.83	
		Median		3.00	
		Variance		4.160	
		Std. Deviation		2.040	
		Minimum		0	
		Maximum		7	
		Range		7	
		Interquartile Range		3	
		Skewness		.234	.350
		Kurtosis		-.979	.688
	Male	Mean		2.76	.392
		95% Confidence Interval for Mean	Lower Bound	1.96	
			Upper Bound	3.55	
		5% Trimmed Mean		2.56	
		Median		2.00	
		Variance		6.289	
		Std. Deviation		2.508	

		Minimum		0	
		Maximum		9	
		Range		9	
		Interquartile Range		4	
		Skewness		1.021	.369
		Kurtosis		.784	.724
BBAQ - Lack of Energy	Female	Mean		2.83	.305
		95% Confidence Interval for Mean	Lower Bound	2.21	
			Upper Bound	3.44	
		5% Trimmed Mean		2.73	
		Median		3.00	
		Variance		4.280	
		Std. Deviation		2.069	
		Minimum		0	
		Maximum		8	
		Range		8	
		Interquartile Range		3	
		Skewness		.668	.350
		Kurtosis		-.100	.688
	Male	Mean		2.20	.388
		95% Confidence Interval for Mean	Lower Bound	1.41	
			Upper Bound	2.98	
		5% Trimmed Mean		2.00	
		Median		1.00	
		Variance		6.161	
		Std. Deviation		2.482	
		Minimum		0	
		Maximum		9	
		Range		9	
		Interquartile Range		4	
		Skewness		1.023	.369
		Kurtosis		.097	.724
BBAQ - Lack of Willpower	Female	Mean		3.11	.342
		95% Confidence Interval for Mean	Lower Bound	2.42	
			Upper Bound	3.80	
		5% Trimmed Mean		2.98	
		Median		3.00	
		Variance		5.388	
		Std. Deviation		2.321	
		Minimum		0	
		Maximum		9	

		Range		9	
		Interquartile Range		3	
		Skewness		.509	.350
		Kurtosis		-.031	.688
	Male	Mean		4.15	.355
		95% Confidence Interval for Mean	Lower Bound	3.43	
			Upper Bound	4.86	
		5% Trimmed Mean		4.14	
		Median		4.00	
		Variance		5.178	
		Std. Deviation		2.276	
		Minimum		0	
		Maximum		9	
		Range		9	
		Interquartile Range		4	
		Skewness		-.056	.369
		Kurtosis		-.686	.724
BBAQ - Fear of Injury	Female	Mean		3.41	.341
		95% Confidence Interval for Mean	Lower Bound	2.73	
			Upper Bound	4.10	
		5% Trimmed Mean		3.32	
		Median		3.00	
		Variance		5.359	
		Std. Deviation		2.315	
		Minimum		0	
		Maximum		9	
		Range		9	
		Interquartile Range		3	
		Skewness		.526	.350
		Kurtosis		-.437	.688
	Male	Mean		2.59	.401
		95% Confidence Interval for Mean	Lower Bound	1.77	
			Upper Bound	3.40	
		5% Trimmed Mean		2.37	
		Median		2.00	
		Variance		6.599	
		Std. Deviation		2.569	
		Minimum		0	
		Maximum		9	
		Range		9	
		Interquartile Range		4	

		Skewness		1.055	.369
		Kurtosis		.418	.724
BBAQ - Lack of Skill	Female	Mean		3.02	.325
		95% Confidence Interval for Mean	Lower Bound	2.37	
			Upper Bound	3.68	
		5% Trimmed Mean		2.93	
		Median		3.00	
		Variance		4.866	
		Std. Deviation		2.206	
		Minimum		0	
		Maximum		9	
		Range		9	
		Interquartile Range		4	
		Skewness		.491	.350
		Kurtosis		-.270	.688
	Male	Mean		3.39	.410
		95% Confidence Interval for Mean	Lower Bound	2.56	
			Upper Bound	4.22	
		5% Trimmed Mean		3.30	
		Median		3.00	
		Variance		6.894	
		Std. Deviation		2.626	
		Minimum		0	
		Maximum		9	
		Range		9	
		Interquartile Range		4	
		Skewness		.492	.369
		Kurtosis		-.713	.724
BBAQ - Lack of Resources	Female	Mean		3.76	.386
		95% Confidence Interval for Mean	Lower Bound	2.98	
			Upper Bound	4.54	
		5% Trimmed Mean		3.69	
		Median		4.00	
		Variance		6.853	
		Std. Deviation		2.618	
		Minimum		0	
		Maximum		9	
		Range		9	
		Interquartile Range		4	
		Skewness		.157	.350
		Kurtosis		-.903	.688
	Male	Mean		4.85	.469

		95% Confidence Interval for Mean	Lower Bound	3.91	
			Upper Bound	5.80	
		5% Trimmed Mean		4.89	
		Median		5.00	
		Variance		9.028	
		Std. Deviation		3.005	
		Minimum		0	
		Maximum		9	
		Range		9	
		Interquartile Range		6	
		Skewness		.043	.369
		Kurtosis		-1.210	.724
GDS Total Score	Female	Mean		4.17	.434
		95% Confidence Interval for Mean	Lower Bound	3.30	
			Upper Bound	5.05	
		5% Trimmed Mean		4.03	
		Median		4.00	
		Variance		8.680	
		Std. Deviation		2.946	
		Minimum		0	
		Maximum		11	
		Range		11	
		Interquartile Range		3	
		Skewness		.776	.350
		Kurtosis		.268	.688
	Male	Mean		4.17	.437
		95% Confidence Interval for Mean	Lower Bound	3.29	
			Upper Bound	5.05	
		5% Trimmed Mean		4.00	
		Median		4.00	
		Variance		7.845	
		Std. Deviation		2.801	
		Minimum		0	
		Maximum		13	
		Range		13	
		Interquartile Range		4	
		Skewness		.813	.369
		Kurtosis		1.088	.724
EQ-5D-3L VAS	Female	Mean		69.78	2.525
		95% Confidence Interval for Mean	Lower Bound	64.70	

			Upper Bound	74.87	
		5% Trimmed Mean		69.76	
		Median		70.00	
		Variance		293.285	
		Std. Deviation		17.126	
		Minimum		40	
		Maximum		100	
		Range		60	
		Interquartile Range		20	
		Skewness		.087	.350
		Kurtosis		-.546	.688
	Male	Mean		69.51	2.776
		95% Confidence Interval for Mean	Lower Bound	63.90	
			Upper Bound	75.12	
		5% Trimmed Mean		69.73	
		Median		70.00	
		Variance		316.006	
		Std. Deviation		17.777	
		Minimum		30	
		Maximum		100	
		Range		70	
		Interquartile Range		28	
		Skewness		-.116	.369
		Kurtosis		-.651	.724
EQ-5D-3L Index Score	Female	Mean		.8333	.01940
		95% Confidence Interval for Mean	Lower Bound	.7943	
			Upper Bound	.8724	
		5% Trimmed Mean		.8424	
		Median		.8330	
		Variance		.017	
		Std. Deviation		.13161	
		Minimum		.45	
		Maximum		1.00	
		Range		.55	
		Interquartile Range		.22	
		Skewness		-.698	.350
		Kurtosis		.869	.688
	Male	Mean		.8551	.02172
		95% Confidence Interval for Mean	Lower Bound	.8112	
			Upper Bound	.8990	

		5% Trimmed Mean		.8704	
		Median		.8440	
		Variance		.019	
		Std. Deviation		.13910	
		Minimum		.31	
		Maximum		1.00	
		Range		.70	
		Interquartile Range		.18	
		Skewness		-1.692	.369
		Kurtosis		5.278	.724
w-FCI Total Score	Female	Mean		4.91	.468
		95% Confidence Interval for Mean	Lower Bound	3.97	
			Upper Bound	5.86	
		5% Trimmed Mean		4.79	
		Median		4.00	
		Variance		10.081	
		Std. Deviation		3.175	
		Minimum		0	
		Maximum		13	
		Range		13	
		Interquartile Range		5	
		Skewness		.527	.350
		Kurtosis		-.305	.688
	Male	Mean		3.00	.342
		95% Confidence Interval for Mean	Lower Bound	2.31	
			Upper Bound	3.69	
		5% Trimmed Mean		2.84	
		Median		3.00	
		Variance		4.800	
		Std. Deviation		2.191	
		Minimum		0	
		Maximum		10	
		Range		10	
		Interquartile Range		3	
		Skewness		.990	.369
		Kurtosis		1.572	.724
No of NCD conditions	Female	Mean		3.46	.289
		95% Confidence Interval for Mean	Lower Bound	2.87	
			Upper Bound	4.04	
		5% Trimmed Mean		3.43	
		Median		3.00	

		Variance		3.854	
		Std. Deviation		1.963	
		Minimum		0	
		Maximum		8	
		Range		8	
		Interquartile Range		3	
		Skewness		.206	.350
		Kurtosis		-.527	.688
	Male	Mean		2.27	.261
		95% Confidence Interval for Mean	Lower Bound	1.74	
			Upper Bound	2.80	
		5% Trimmed Mean		2.16	
		Median		2.00	
		Variance		2.801	
		Std. Deviation		1.674	
		Minimum		0	
		Maximum		7	
		Range		7	
		Interquartile Range		2	
		Skewness		.796	.369
		Kurtosis		.574	.724

a. Location 1 = Urban, 2 = Rural = Rural

Percentiles[a]

		Biological Sex F = 1 M = 2	Percentiles 25	50	75
Weighted Average(Definition 1)	Age - Years	Female	72.00	76.50	82.00
		Male	65.00	72.00	78.50
	Resting Heart Rate (beats per minute)	Female	77.75	82.00	88.00
		Male	70.00	78.00	88.00
	Resting Systolic Blood Pressure (mmHg)	Female	129.00	133.00	139.00
		Male	119.00	128.00	135.50
	Resting Diastolic Blood Pressure (mmHg)	Female	77.00	80.50	89.00
		Male	73.00	78.00	84.00
	Resting Oxygen Saturation % (SPO2)	Female	98.00	98.00	99.00
		Male	98.00	98.00	99.00
	BMI kg/m2	Female	23.1756	25.9504	31.2598
		Male	24.4182	26.0790	29.9161
	Nutritional Status - 0 = Underweight, 1 = Normal, 2 = Overweight, 3 = Obese	Female	1.00	2.00	3.00
		Male	1.00	2.00	2.50
	Hand Grip Strength	Female	10.2750	12.6000	15.0000
		Male	15.7500	25.8000	30.4000
	Hip-waist-ratio (kg)	Female	.8646	.8992	.9517
		Male	.8618	.8992	.9761
	SPBB - Total score	Female	4.00	6.00	8.00
		Male	5.00	7.00	9.00
	Vigrous MET/week	Female	.0000	.0000	.0000
		Male	.0000	.0000	.0000
	Moderate MET/week	Female	.0000	.0000	150.0000

		Male	.0000	.0000	340.0000
	Walking MET/week	Female	231.0000	643.5000	1386.0000
		Male	462.0000	693.0000	1386.0000
	IPAQ - No. of minutes spent sitting per day in last 7 days	Female	120.00	180.00	180.00
		Male	120.00	180.00	180.00
	Total MET/week	Female	349.8750	939.0000	1665.7500
		Male	516.0000	1039.5000	1386.0000
	BBAQ - Lack of time	Female	1.00	2.00	4.25
		Male	.00	1.00	4.50
	BBAQ - Social Influence	Female	1.00	3.00	4.25
		Male	.50	2.00	4.00
	BBAQ - Lack of Energy	Female	1.00	3.00	4.00
		Male	.00	1.00	4.00
	BBAQ - Lack of Willpower	Female	1.00	3.00	4.25
		Male	2.00	4.00	6.00
	BBAQ - Fear of Injury	Female	2.00	3.00	5.25
		Male	.50	2.00	4.00
	BBAQ - Lack of Skill	Female	1.00	3.00	5.00
		Male	1.00	3.00	5.00
	BBAQ - Lack of Resources	Female	1.75	4.00	6.00
		Male	2.50	5.00	8.00
	GDS Total Score	Female	1.75	4.00	5.00
		Male	2.00	4.00	6.00
	EQ-5D-3L VAS	Female	60.00	70.00	80.00
		Male	52.50	70.00	80.00
	EQ-5D-3L Index Score	Female	.7770	.8330	1.0000
		Male	.8170	.8440	1.0000
	w-FCI Total Score	Female	2.00	4.00	7.25
		Male	1.50	3.00	4.00
	No of NCD conditions	Female	2.00	3.00	5.00
		Male	1.00	2.00	3.00
Tukey's Hinges	Age - Years	Female	72.00	76.50	82.00
		Male	65.00	72.00	78.00
	Resting Heart Rate (beats per minute)	Female	78.00	82.00	88.00
		Male	71.00	78.00	88.00
	Resting Systolic Blood Pressure (mmHg)	Female	129.00	133.00	139.00
		Male	120.00	128.00	135.00
	Resting Diastolic Blood Pressure (mmHg)	Female	77.00	80.50	89.00
		Male	74.00	78.00	84.00
	Resting Oxygen Saturation % (SPO2)	Female	98.00	98.00	99.00
		Male	98.00	98.00	99.00
	BMI kg/m2	Female	23.1972	25.9504	31.2500
		Male	24.5890	26.0790	29.7705
	Nutritional Status - 0 = Underweight, 1 = Normal, 2 = Overweight, 3 = Obese	Female	1.00	2.00	3.00
		Male	1.00	2.00	2.00
	Hand Grip Strength	Female	10.3000	12.6000	15.0000
		Male	16.4000	25.8000	30.0000
	Hip-waist-ratio (kg)	Female	.8648	.8992	.9501
		Male	.8636	.8992	.9684
	SPBB - Total score	Female	4.00	6.00	8.00
		Male	5.00	7.00	9.00
	Vigrous MET/week	Female	.0000	.0000	.0000
		Male	.0000	.0000	.0000
	Moderate MET/week	Female	.0000	.0000	120.0000
		Male	.0000	.0000	320.0000
	Walking MET/week	Female	231.0000	643.5000	1386.0000
		Male	462.0000	693.0000	1386.0000
	IPAQ - No. of minutes spent sitting per day in last 7 days	Female	120.00	180.00	180.00
		Male	120.00	180.00	180.00
	Total MET/week	Female	351.0000	939.0000	1533.0000
		Male	537.0000	1039.5000	1386.0000

	BBAQ - Lack of time	Female	1.00	2.00	4.00
		Male	.00	1.00	4.00
	BBAQ - Social Influence	Female	1.00	3.00	4.00
		Male	1.00	2.00	4.00
	BBAQ - Lack of Energy	Female	1.00	3.00	4.00
		Male	.00	1.00	4.00
	BBAQ - Lack of Willpower	Female	1.00	3.00	4.00
		Male	2.00	4.00	6.00
	BBAQ - Fear of Injury	Female	2.00	3.00	5.00
		Male	1.00	2.00	4.00
	BBAQ - Lack of Skill	Female	1.00	3.00	5.00
		Male	1.00	3.00	5.00
	BBAQ - Lack of Resources	Female	2.00	4.00	6.00
		Male	3.00	5.00	8.00
	GDS Total Score	Female	2.00	4.00	5.00
		Male	2.00	4.00	6.00
	EQ-5D-3L VAS	Female	60.00	70.00	80.00
		Male	55.00	70.00	80.00
	EQ-5D-3L Index Score	Female	.7770	.8330	1.0000
		Male	.8330	.8440	1.0000
	w-FCI Total Score	Female	2.00	4.00	7.00
		Male	2.00	3.00	4.00
	No of NCD conditions	Female	2.00	3.00	5.00
		Male	1.00	2.00	3.00

a. Location 1 = Urban, 2 = Rural = Rural

1.1.2 Full sample descriptive statistics

Case Processing Summary						
	Cases					
	Valid		Missing		Total	
	N	Percent	N	Percent	N	Percent
Age - Years	396	100.0%	0	0.0%	396	100.0%
Resting Heart Rate (beats per minute)	396	100.0%	0	0.0%	396	100.0%
Resting Systolic Blood Pressure (mmHg)	396	100.0%	0	0.0%	396	100.0%
Resting Diastolic Blood Pressure (mmHg)	396	100.0%	0	0.0%	396	100.0%
Resting Oxygen Saturation % (SPO2)	396	100.0%	0	0.0%	396	100.0%
BMI kg/m2	396	100.0%	0	0.0%	396	100.0%
Nutritional Status - 0 = Underweight, 1 = Normal, 2 = Overweight, 3 = Obese	396	100.0%	0	0.0%	396	100.0%
Hand Grip Strength	396	100.0%	0	0.0%	396	100.0%
Hip-waist-ratio (kg)	396	100.0%	0	0.0%	396	100.0%
SPBB - Total score	396	100.0%	0	0.0%	396	100.0%
Vigrous MET/week	396	100.0%	0	0.0%	396	100.0%
Moderate MET/week	396	100.0%	0	0.0%	396	100.0%
Walking MET/week	396	100.0%	0	0.0%	396	100.0%
IPAQ - No. of minutes spent sitting per day in last 7 days	396	100.0%	0	0.0%	396	100.0%
Total MET/week	396	100.0%	0	0.0%	396	100.0%
BBAQ - Lack of time	396	100.0%	0	0.0%	396	100.0%
BBAQ - Social Influence	396	100.0%	0	0.0%	396	100.0%
BBAQ - Lack of Energy	396	100.0%	0	0.0%	396	100.0%
BBAQ - Lack of Willpower	396	100.0%	0	0.0%	396	100.0%
BBAQ - Fear of Injury	396	100.0%	0	0.0%	396	100.0%

BBAQ - Lack of Skill	396	100.0%	0	0.0%	396	100.0%
BBAQ - Lack of Resources	396	100.0%	0	0.0%	396	100.0%
GDS Total Score	396	100.0%	0	0.0%	396	100.0%
EQ-5D-3L VAS	396	100.0%	0	0.0%	396	100.0%
EQ-5D-3L Index Score	396	100.0%	0	0.0%	396	100.0%
w-FCI Total Score	396	100.0%	0	0.0%	396	100.0%
No of NCD conditions	396	100.0%	0	0.0%	396	100.0%

Descriptives

			Statistic	Std. Error
Age - Years	Mean		72.87	.387
	95% Confidence Interval for Mean	Lower Bound	72.11	
		Upper Bound	73.63	
	5% Trimmed Mean		72.63	
	Median		72.50	
	Variance		59.319	
	Std. Deviation		7.702	
	Minimum		56	
	Maximum		98	
	Range		42	
	Interquartile Range		11	
	Skewness		.382	.123
	Kurtosis		-.267	.245
Resting Heart Rate (beats per minute)	Mean		78.89	.543
	95% Confidence Interval for Mean	Lower Bound	77.82	
		Upper Bound	79.95	
	5% Trimmed Mean		79.01	
	Median		79.00	
	Variance		116.911	
	Std. Deviation		10.813	
	Minimum		51	
	Maximum		100	
	Range		49	
	Interquartile Range		17	
	Skewness		-.164	.123
	Kurtosis		-.563	.245
Resting Systolic Blood Pressure (mmHg)	Mean		129.60	.465
	95% Confidence Interval for Mean	Lower Bound	128.69	
		Upper Bound	130.52	
	5% Trimmed Mean		130.24	
	Median		132.00	
	Variance		85.501	
	Std. Deviation		9.247	
	Minimum		92	

	Maximum		140	
	Range		48	
	Interquartile Range		15	
	Skewness		-.890	.123
	Kurtosis		.230	.245
Resting Diastolic Blood Pressure (mmHg)	Mean		77.16	.477
	95% Confidence Interval for Mean	Lower Bound	76.22	
		Upper Bound	78.09	
	5% Trimmed Mean		77.35	
	Median		78.00	
	Variance		90.193	
	Std. Deviation		9.497	
	Minimum		52	
	Maximum		100	
	Range		48	
	Interquartile Range		13	
	Skewness		-.263	.123
	Kurtosis		-.364	.245
Resting Oxygen Saturation % (SPO2)	Mean		97.72	.050
	95% Confidence Interval for Mean	Lower Bound	97.63	
		Upper Bound	97.82	
	5% Trimmed Mean		97.76	
	Median		98.00	
	Variance		1.005	
	Std. Deviation		1.003	
	Minimum		95	
	Maximum		99	
	Range		4	
	Interquartile Range		1	
	Skewness		-.654	.123
	Kurtosis		-.409	.245
BMI kg/m2	Mean		27.5288	.27591
	95% Confidence Interval for Mean	Lower Bound	26.9864	
		Upper Bound	28.0712	
	5% Trimmed Mean		27.3992	
	Median		26.6772	
	Variance		30.146	
	Std. Deviation		5.49052	
	Minimum		15.21	
	Maximum		41.62	
	Range		26.42	
	Interquartile Range		7.91	

	Skewness		.353	.123
	Kurtosis		-.416	.245
Nutritional Status - 0 = Underweight, 1 = Normal, 2 = Overweight, 3 = Obese	Mean		1.93	.043
	95% Confidence Interval for Mean	Lower Bound	1.85	
		Upper Bound	2.02	
	5% Trimmed Mean		1.95	
	Median		2.00	
	Variance		.747	
	Std. Deviation		.864	
	Minimum		0	
	Maximum		3	
	Range		3	
	Interquartile Range		2	
	Skewness		-.081	.123
	Kurtosis		-1.222	.245
Hand Grip Strength	Mean		16.6381	.40171
	95% Confidence Interval for Mean	Lower Bound	15.8484	
		Upper Bound	17.4279	
	5% Trimmed Mean		16.0479	
	Median		14.9000	
	Variance		63.902	
	Std. Deviation		7.99388	
	Minimum		2.40	
	Maximum		45.90	
	Range		43.50	
	Interquartile Range		9.58	
	Skewness		1.194	.123
	Kurtosis		1.393	.245
Hip-waist-ratio (kg)	Mean		.9322	.00627
	95% Confidence Interval for Mean	Lower Bound	.9198	
		Upper Bound	.9445	
	5% Trimmed Mean		.9221	
	Median		.9071	
	Variance		.016	
	Std. Deviation		.12486	
	Minimum		.64	
	Maximum		2.15	
	Range		1.50	
	Interquartile Range		.11	
	Skewness		3.613	.123
	Kurtosis		26.696	.245
SPBB - Total score	Mean		6.72	.161
	95% Confidence Interval for Mean	Lower Bound	6.40	

		Upper Bound	7.04	
	5% Trimmed Mean		6.77	
	Median		7.00	
	Variance		10.314	
	Std. Deviation		3.211	
	Minimum		0	
	Maximum		12	
	Range		12	
	Interquartile Range		5	
	Skewness		-.214	.123
	Kurtosis		-.850	.245
Vigrous MET/week	Mean		185.3030	46.67410
	95% Confidence Interval for Mean	Lower Bound	93.5423	
		Upper Bound	277.0637	
	5% Trimmed Mean		24.0741	
	Median		.0000	
	Variance		862674.591	
	Std. Deviation		928.80277	
	Minimum		.00	
	Maximum		10080.00	
	Range		10080.00	
	Interquartile Range		.00	
	Skewness		7.677	.123
	Kurtosis		70.193	.245
Moderate MET/week	Mean		415.1111	37.73505
	95% Confidence Interval for Mean	Lower Bound	340.9245	
		Upper Bound	489.2978	
	5% Trimmed Mean		290.9315	
	Median		.0000	
	Variance		563877.851	
	Std. Deviation		750.91801	
	Minimum		.00	
	Maximum		5040.00	
	Range		5040.00	
	Interquartile Range		540.00	
	Skewness		3.046	.123
	Kurtosis		11.058	.245
Walking MET/week	Mean		1103.8750	56.46587
	95% Confidence Interval for Mean	Lower Bound	992.8638	
		Upper Bound	1214.8862	
	5% Trimmed Mean		995.5278	
	Median		693.0000	

	Variance		1262604.216	
	Std. Deviation		1123.65663	
	Minimum		.00	
	Maximum		4158.00	
	Range		4158.00	
	Interquartile Range		1089.00	
	Skewness		1.411	.123
	Kurtosis		1.234	.245
IPAQ - No. of minutes spent sitting per day in last 7 days	Mean		152.59	2.292
	95% Confidence Interval for Mean	Lower Bound	148.08	
		Upper Bound	157.10	
	5% Trimmed Mean		158.08	
	Median		180.00	
	Variance		2081.195	
	Std. Deviation		45.620	
	Minimum		10	
	Maximum		180	
	Range		170	
	Interquartile Range		60	
	Skewness		-1.558	.123
	Kurtosis		1.369	.245
Total MET/week	Mean		1611.0177	72.61258
	95% Confidence Interval for Mean	Lower Bound	1468.2622	
		Upper Bound	1753.7731	
	5% Trimmed Mean		1504.4534	
	Median		1053.0000	
	Variance		2087944.466	
	Std. Deviation		1444.97213	
	Minimum		20.00	
	Maximum		5172.00	
	Range		5152.00	
	Interquartile Range		1646.25	
	Skewness		1.067	.123
	Kurtosis		-.098	.245
BBAQ - Lack of time	Mean		2.77	.133
	95% Confidence Interval for Mean	Lower Bound	2.51	
		Upper Bound	3.03	
	5% Trimmed Mean		2.59	
	Median		2.00	
	Variance		6.966	
	Std. Deviation		2.639	
	Minimum		0	

	Maximum		9	
	Range		9	
	Interquartile Range		5	
	Skewness		.688	.123
	Kurtosis		-.565	.245
BBAQ - Social Influence	Mean		3.00	.133
	95% Confidence Interval for Mean	Lower Bound	2.74	
		Upper Bound	3.26	
	5% Trimmed Mean		2.83	
	Median		3.00	
	Variance		7.030	
	Std. Deviation		2.651	
	Minimum		0	
	Maximum		9	
	Range		9	
	Interquartile Range		5	
	Skewness		.670	.123
	Kurtosis		-.424	.245
BBAQ - Lack of Energy	Mean		2.73	.127
	95% Confidence Interval for Mean	Lower Bound	2.48	
		Upper Bound	2.98	
	5% Trimmed Mean		2.54	
	Median		2.00	
	Variance		6.412	
	Std. Deviation		2.532	
	Minimum		0	
	Maximum		9	
	Range		9	
	Interquartile Range		4	
	Skewness		.764	.123
	Kurtosis		-.242	.245
BBAQ - Lack of Willpower	Mean		3.70	.161
	95% Confidence Interval for Mean	Lower Bound	3.39	
		Upper Bound	4.02	
	5% Trimmed Mean		3.53	
	Median		3.00	
	Variance		10.214	
	Std. Deviation		3.196	
	Minimum		0	
	Maximum		39	
	Range		39	
	Interquartile Range		5	

	Skewness		3.559	.123
	Kurtosis		36.235	.245
BBAQ - Fear of Injury	Mean		2.96	.131
	95% Confidence Interval for Mean	Lower Bound	2.70	
		Upper Bound	3.21	
	5% Trimmed Mean		2.80	
	Median		3.00	
	Variance		6.801	
	Std. Deviation		2.608	
	Minimum		0	
	Maximum		9	
	Range		9	
	Interquartile Range		4	
	Skewness		.624	.123
	Kurtosis		-.556	.245
BBAQ - Lack of Skill	Mean		3.08	.131
	95% Confidence Interval for Mean	Lower Bound	2.82	
		Upper Bound	3.33	
	5% Trimmed Mean		2.92	
	Median		3.00	
	Variance		6.751	
	Std. Deviation		2.598	
	Minimum		0	
	Maximum		9	
	Range		9	
	Interquartile Range		4	
	Skewness		.642	.123
	Kurtosis		-.466	.245
BBAQ - Lack of Resources	Mean		4.64	.145
	95% Confidence Interval for Mean	Lower Bound	4.35	
		Upper Bound	4.92	
	5% Trimmed Mean		4.65	
	Median		5.00	
	Variance		8.378	
	Std. Deviation		2.894	
	Minimum		0	
	Maximum		9	
	Range		9	
	Interquartile Range		5	
	Skewness		-.046	.123
	Kurtosis		-1.127	.245
GDS Total Score	Mean		3.81	.150
	95% Confidence Interval for Mean	Lower Bound	3.52	

		Upper Bound	4.11	
	5% Trimmed Mean		3.61	
	Median		3.00	
	Variance		8.888	
	Std. Deviation		2.981	
	Minimum		0	
	Maximum		14	
	Range		14	
	Interquartile Range		4	
	Skewness		.945	.123
	Kurtosis		.379	.245
EQ-5D-3L VAS	Mean		70.46	.855
	95% Confidence Interval for Mean	Lower Bound	68.78	
		Upper Bound	72.14	
	5% Trimmed Mean		70.81	
	Median		70.00	
	Variance		289.495	
	Std. Deviation		17.015	
	Minimum		20	
	Maximum		100	
	Range		80	
	Interquartile Range		20	
	Skewness		-.248	.123
	Kurtosis		-.410	.245
EQ-5D-3L Index Score	Mean		.8307	.00705
	95% Confidence Interval for Mean	Lower Bound	.8168	
		Upper Bound	.8446	
	5% Trimmed Mean		.8414	
	Median		.8330	
	Variance		.020	
	Std. Deviation		.14030	
	Minimum		.27	
	Maximum		1.00	
	Range		.73	
	Interquartile Range		.22	
	Skewness		-.855	.123
	Kurtosis		1.242	.245
w-FCI Total Score	Mean		4.34	.163
	95% Confidence Interval for Mean	Lower Bound	4.02	
		Upper Bound	4.66	
	5% Trimmed Mean		4.11	
	Median		4.00	

	Variance		10.554	
	Std. Deviation		3.249	
	Minimum		0	
	Maximum		19	
	Range		19	
	Interquartile Range		4	
	Skewness		1.096	.123
	Kurtosis		1.754	.245
No of NCD conditions	Mean		3.04	.100
	95% Confidence Interval for Mean	Lower Bound	2.84	
		Upper Bound	3.24	
	5% Trimmed Mean		2.95	
	Median		3.00	
	Variance		3.973	
	Std. Deviation		1.993	
	Minimum		0	
	Maximum		10	
	Range		10	
	Interquartile Range		2	
	Skewness		.617	.123
	Kurtosis		.038	.245

Percentiles

		Percentiles		
		25	50	75
Weighted Average(Definition 1)	Age - Years	67.00	72.50	78.00
	Resting Heart Rate (beats per minute)	71.00	79.00	88.00
	Resting Systolic Blood Pressure (mmHg)	123.00	132.00	138.00
	Resting Diastolic Blood Pressure (mmHg)	71.00	78.00	84.00
	Resting Oxygen Saturation % (SPO2)	97.00	98.00	98.00
	BMI kg/m2	23.3794	26.6772	31.2876
	Nutritional Status - 0 = Underweight, 1 = Normal, 2 = Overweight, 3 = Obese	1.00	2.00	3.00
	Hand Grip Strength	10.8000	14.9000	20.3750
	Hip-waist-ratio (kg)	.8647	.9071	.9706
	SPBB - Total score	4.25	7.00	9.00
	Vigrous MET/week	.0000	.0000	.0000
	Moderate MET/week	.0000	.0000	540.0000
	Walking MET/week	297.0000	693.0000	1386.0000
	IPAQ - No. of minutes spent sitting per day in last 7 days	120.00	180.00	180.00
	Total MET/week	480.0000	1053.0000	2126.2500
	BBAQ - Lack of time	.00	2.00	5.00
	BBAQ - Social Influence	.25	3.00	5.00
	BBAQ - Lack of Energy	.00	2.00	4.00
	BBAQ - Lack of Willpower	1.00	3.00	6.00
	BBAQ - Fear of Injury	1.00	3.00	5.00
	BBAQ - Lack of Skill	1.00	3.00	5.00
	BBAQ - Lack of Resources	2.00	5.00	7.00
	GDS Total Score	1.00	3.00	5.00
	EQ-5D-3L VAS	60.00	70.00	80.00
	EQ-5D-3L Index Score	.7770	.8330	1.0000
	w-FCI Total Score	2.00	4.00	6.00
	No of NCD conditions	2.00	3.00	4.00
Tukey's Hinges	Age - Years	67.00	72.50	78.00
	Resting Heart Rate (beats per minute)	71.00	79.00	88.00
	Resting Systolic Blood Pressure (mmHg)	123.00	132.00	138.00
	Resting Diastolic Blood Pressure (mmHg)	71.00	78.00	84.00

	Resting Oxygen Saturation % (SPO2)	97.00	98.00	98.00
	BMI kg/m2	23.3811	26.6772	31.2862
	Nutritional Status - 0 = Underweight, 1 = Normal, 2 = Overweight, 3 = Obese	1.00	2.00	3.00
	Hand Grip Strength	10.8000	14.9000	20.3500
	Hip-waist-ratio (kg)	.8647	.9071	.9705
	SPBB - Total score	4.50	7.00	9.00
	Vigrous MET/week	.0000	.0000	.0000
	Moderate MET/week	.0000	.0000	540.0000
	Walking MET/week	297.0000	693.0000	1386.0000
	IPAQ - No. of minutes spent sitting per day in last 7 days	120.00	180.00	180.00
	Total MET/week	480.0000	1053.0000	2119.5000
	BBAQ - Lack of time	.00	2.00	5.00
	BBAQ - Social Influence	.50	3.00	5.00
	BBAQ - Lack of Energy	.00	2.00	4.00
	BBAQ - Lack of Willpower	1.00	3.00	6.00
	BBAQ - Fear of Injury	1.00	3.00	5.00
	BBAQ - Lack of Skill	1.00	3.00	5.00
	BBAQ - Lack of Resources	2.00	5.00	7.00
	GDS Total Score	1.00	3.00	5.00
	EQ-5D-3L VAS	60.00	70.00	80.00
	EQ-5D-3L Index Score	.7770	.8330	1.0000
	w-FCI Total Score	2.00	4.00	6.00
	No of NCD conditions	2.00	3.00	4.00

1.2 BMI Linear regression model

ANOVA

Model		Sum of Squares	df	Mean Square	F	Sig.
1	Regression	2139.866	12	178.322	6.992	<.001[b]
	Residual	9767.749	383	25.503		
	Total	11907.615	395			

a. Dependent Variable: BMI kg/m2

b. Predictors: (Constant), SPPB_0, GDS_2, Walking MET/week, Hip-waist-ratio (kg), Resting Systolic Blood Pressure (mmHg), BBAQ_Resou_8, w_FCI_9, IPAQ - No. of minutes spent sitting per day in last 7 days , BBAQ_Will_6, Age - Years , w-FCI Arthritis N = 0, Y = 0 (no influence), 1 (Partial Influence), 2 (Severe Influence), Balance_DUM_0

Coefficients

Model		Unstandardized Coefficients		Standardized Coefficients	t	Sig.	Collinearity Statistics	
		B	Std. Error	Beta			Tolerance	VIF
1	(Constant)	17.973	4.873		3.688	<.001		

	Balance_DUM_0	2.857	.746	.191	3.832	<.001	.866	1.155
	Resting Systolic Blood Pressure (mmHg)	.076	.028	.129	2.746	.006	.978	1.023
	GDS_2	-2.010	.690	-.137	-2.912	.004	.962	1.040
	Age - Years	-.103	.034	-.145	-3.040	.003	.946	1.057
	w_FCI_9	2.814	1.190	.112	2.366	.018	.949	1.054
	Hip-waist-ratio (kg)	5.767	2.101	.131	2.744	.006	.938	1.066
	Walking MET/week	-.001	.000	-.126	-2.664	.008	.960	1.042
	IPAQ - No. of minutes spent sitting per day in last 7 days	.013	.006	.105	2.233	.026	.966	1.036
	w-FCI Arthritis	.795	.339	.112	2.344	.020	.938	1.066
	BBAQ_Resources	2.670	1.146	.109	2.329	.020	.976	1.025
	BBAQ_Willpower	-1.757	.833	-.100	-2.111	.035	.960	1.042
	SPPB_0	-3.759	1.956	-.096	-1.921	.055	.850	1.176

a. Dependent Variable: BMI kg/m2

Residuals Statistics

	Minimum	Maximum	Mean	Std. Deviation	N
Predicted Value	20.8197	35.0114	27.5288	2.32753	396
Residual	-12.10604	13.76933	.00000	4.97277	396
Std. Predicted Value	-2.883	3.215	.000	1.000	396
Std. Residual	-2.397	2.727	.000	.985	396

a. Dependent Variable: BMI kg/m2

1.3 Geriatric Depression Scale regression model

Model Fitting Information

Model	-2 Log Likelihood	Chi-Square	df	Sig.
Intercept Only	1846.998			
Final	1467.118	379.880	158	<.001

Pseudo R-Square

Cox and Snell	.617
Nagelkerke	.623
McFadden	.206

Link function: Logit.

Parameter Estimates

		Estimate	Std. Error	Wald	df	Sig.	95% Confidence Interval	
							Lower Bound	Upper Bound
Threshold	[GDSTotalScore = 0]	13.243	18.274	.525	1	.469	-22.573	49.058
	[GDSTotalScore = 1]	15.089	18.275	.682	1	.409	-20.730	50.907
	[GDSTotalScore = 2]	16.391	18.276	.804	1	.370	-19.430	52.213
	[GDSTotalScore = 3]	17.262	18.277	.892	1	.345	-18.560	53.085
	[GDSTotalScore = 4]	18.097	18.278	.980	1	.322	-17.727	53.921
	[GDSTotalScore = 5]	19.072	18.281	1.088	1	.297	-16.757	54.902
	[GDSTotalScore = 6]	19.886	18.284	1.183	1	.277	-15.951	55.723
	[GDSTotalScore = 7]	20.495	18.287	1.256	1	.262	-15.347	56.337
	[GDSTotalScore = 8]	21.266	18.290	1.352	1	.245	-14.581	57.114
	[GDSTotalScore = 9]	21.732	18.292	1.412	1	.235	-14.119	57.582
	[GDSTotalScore = 10]	22.507	18.295	1.513	1	.219	-13.350	58.364
	[GDSTotalScore = 11]	24.799	18.318	1.833	1	.176	-11.103	60.701
	[GDSTotalScore = 12]	25.167	18.324	1.887	1	.170	-10.746	61.081
	[GDSTotalScore = 13]	26.347	18.352	2.061	1	.151	-9.621	62.316
Location	AgeYears	.025	.017	2.161	1	.142	-.008	.058
	RestingHeartRatebeatsperminute	.019	.011	3.047	1	.081	-.002	.040
	RestingSystolicBloodPressuremmHg	-.013	.013	.948	1	.330	-.038	.013
	RestingDiastolicBloodPressuremmHg	.001	.013	.002	1	.961	-.026	.027
	RestingOxygenSaturationSPO2	.008	.122	.005	1	.946	-.231	.247
	BMIkgm2	.100	.037	7.250	1	.007	.027	.172
	HandGripStrength	.003	.018	.028	1	.867	-.032	.038
	Hipwaistratiokg	-1.343	.999	1.809	1	.179	-3.301	.614
	VigrousMETweek	4.648E-5	.000	.093	1	.761	.000	.000
	ModerateMETweek	-9.862E-5	.000	.124	1	.725	-.001	.000
	WalkingMETweek	.000	.000	.435	1	.510	-.001	.000
	IPAQNo.ofminutesspentsittingperdayinlast7days	.006	.003	5.230	1	.022	.001	.011
	TotalMETweek	.000	.000	.391	1	.532	.000	.001
	EQ5D3LVAS	-.026	.007	12.059	1	<.001	-.040	-.011
	EQ5D3LIndexScore	4.777	3.452	1.915	1	.166	-1.989	11.543
	[Location1Urban2Rural=1]	-.487	.309	2.482	1	.115	-1.092	.119
	[Location1Urban2Rural=2]	0[a]	.	.	0	.	.	.
	[BiologicalSexF1M2=1]	-.827	.293	7.987	1	.005	-1.401	-.254
	[BiologicalSexF1M2=2]	0[a]	.	.	0	.	.	.
	[ArthritisN0Y1=0]	.460	.354	1.683	1	.194	-.235	1.154
	[ArthritisN0Y1=1]	0[a]	.	.	0	.	.	.
	[OsteoporosisN0Y1=0]	1.231	.408	9.082	1	.003	.430	2.031
	[OsteoporosisN0Y1=1]	0[a]	.	.	0	.	.	.
	[DegenerativeDiscN0Y1=0]	.996	.373	7.136	1	.008	.265	1.727
	[DegenerativeDiscN0Y1=1]	0[a]	.	.	0	.	.	.
	[PulmonaryDxN0Y1=0]	.292	.390	.561	1	.454	-.472	1.055
	[PulmonaryDxN0Y1=1]	0[a]	.	.	0	.	.	.
	[AnginaPectorisN0Y1=0]	-.252	.522	.233	1	.629	-1.276	.772
	[AnginaPectorisN0Y1=1]	0[a]	.	.	0	.	.	.

[MyocardialInfarctionN0Y1=0]	1.175	.745	2.490	1	.115	-.285	2.635
[MyocardialInfarctionN0Y1=1]	0[a]	.	.	0	.	.	.
[HeartFailureN0Y1=0]	1.036	.688	2.270	1	.132	-.312	2.384
[HeartFailureN0Y1=1]	0[a]	.	.	0	.	.	.
[NeurologicalDxN0Y1=0]	.238	.635	.140	1	.708	-1.006	1.482
[NeurologicalDxN0Y1=1]	0[a]	.	.	0	.	.	.
[DementiaN0Y1=0]	0[a]	.	.	0	.	.	.
[CVADxN0Y1=0]	.495	.573	.749	1	.387	-.627	1.618
[CVADxN0Y1=1]	0[a]	.	.	0	.	.	.
[PeripheralVascularDxN0Y1=0]	.937	.469	3.990	1	.046	.018	1.857
[PeripheralVascularDxN0Y1=1]	0[a]	.	.	0	.	.	.
[DiabetesN0Y1=0]	.558	.364	2.357	1	.125	-.154	1.271
[DiabetesN0Y1=1]	0[a]	.	.	0	.	.	.
[GastrointestinalDxN0Y1=0]	.422	.437	.932	1	.334	-.434	1.277
[GastrointestinalDxN0Y1=1]	0[a]	.	.	0	.	.	.
[Obesity=0]	.906	.420	4.655	1	.031	.083	1.730
[Obesity=1]	0[a]	.	.	0	.	.	.
[VisualImpairmentN0Y1=0]	.851	.364	5.460	1	.019	.137	1.564
[VisualImpairmentN0Y1=1]	0[a]	.	.	0	.	.	.
[HearingImpairmentN0Y1=0]	1.032	.907	1.295	1	.255	-.746	2.810
[HearingImpairmentN0Y1=1]	0[a]	.	.	0	.	.	.
[NoofNCDconditions=0]	-8.860	3.553	6.219	1	.013	-15.824	-1.897
[NoofNCDconditions=1]	-8.667	3.366	6.631	1	.010	-15.264	-2.070
[NoofNCDconditions=2]	-8.229	3.223	6.518	1	.011	-14.546	-1.912
[NoofNCDconditions=3]	-8.236	3.061	7.240	1	.007	-14.235	-2.237
[NoofNCDconditions=4]	-7.834	2.927	7.165	1	.007	-13.570	-2.098
[NoofNCDconditions=5]	-5.497	2.805	3.842	1	.050	-10.994	-9.851E-5
[NoofNCDconditions=6]	-6.568	2.720	5.829	1	.016	-11.899	-1.236
[NoofNCDconditions=7]	-5.682	2.692	4.457	1	.035	-10.957	-.407
[NoofNCDconditions=8]	-2.170	2.623	.685	1	.408	-7.310	2.970
[NoofNCDconditions=9]	4.999	3.416	2.141	1	.143	-1.697	11.694
[NoofNCDconditions=10]	0[a]	.	.	0	.	.	.
[Comorbidities=0]	0[a]	.	.	0	.	.	.
[Comorbidities=1]	0[a]	.	.	0	.	.	.
[SingleDUM=.000]	.130	.356	.134	1	.715	-.568	.828
[SingleDUM=1.000]	0[a]	.	.	0	.	.	.
[MarriedDUM=.000]	.012	.364	.001	1	.973	-.700	.725
[MarriedDUM=1.000]	0[a]	.	.	0	.	.	.
[WidowDUM=.000]	.339	.336	1.017	1	.313	-.320	.998
[WidowDUM=1.000]	0[a]	.	.	0	.	.	.
[DivorcedDUM=.000]	0[a]	.	.	0	.	.	.
[DivorcedDUM=1.000]	0[a]	.	.	0	.	.	.

[No_ED_DUMMY=.000]	2.415	.878	7.566	1	.006	.694	4.136
[No_ED_DUMMY=1.000]	0[a]	.	.	0	.	.	.
[Prim_ED_DUMMY=.000]	.630	.700	.812	1	.368	-.741	2.001
[Prim_ED_DUMMY=1.000]	0[a]	.	.	0	.	.	.
[High_ED_DUMMY=.000]	1.329	.642	4.286	1	.038	.071	2.587
[High_ED_DUMMY=1.000]	0[a]	.	.	0	.	.	.
[Diploma_ED_DUMMY=.000]	.587	.748	.616	1	.433	-.879	2.054
[Diploma_ED_DUMMY=1.000]	0[a]	.	.	0	.	.	.
[Degree_DUMMY=.000]	0[a]	.	.	0	.	.	.
[Degree_DUMMY=1.000]	0[a]	.	.	0	.	.	.
[Balance_DUM_0=.000]	-1.564	.775	4.073	1	.044	-3.082	-.045
[Balance_DUM_0=1.000]	0[a]	.	.	0	.	.	.
[Balance_DUM_1=.000]	-1.578	.568	7.718	1	.005	-2.691	-.465
[Balance_DUM_1=1.000]	0[a]	.	.	0	.	.	.
[Balance_DUM_2=.000]	-.295	.466	.401	1	.527	-1.209	.619
[Balance_DUM_2=1.000]	0[a]	.	.	0	.	.	.
[Balance_DUM_3=.000]	-.334	.406	.675	1	.411	-1.130	.462
[Balance_DUM_3=1.000]	0[a]	.	.	0	.	.	.
[Balance_DUM_4=.000]	0[a]	.	.	0	.	.	.
[Balance_DUM_4=1.000]	0[a]	.	.	0	.	.	.
[Gait_DUM_0=.000]	-2.495	1.269	3.865	1	.049	-4.982	-.008
[Gait_DUM_0=1.000]	0[a]	.	.	0	.	.	.
[Gait_DUM_1=.000]	-1.647	.447	13.591	1	<.001	-2.522	-.771
[Gait_DUM_1=1.000]	0[a]	.	.	0	.	.	.
[Gait_DUM_2=.000]	-.482	.411	1.379	1	.240	-1.287	.323
[Gait_DUM_2=1.000]	0[a]	.	.	0	.	.	.
[Gait_DUM_3=.000]	.062	.366	.029	1	.866	-.656	.780
[Gait_DUM_3=1.000]	0[a]	.	.	0	.	.	.
[Gait_DUM_4=.000]	0[a]	.	.	0	.	.	.
[Gait_DUM_4=1.000]	0[a]	.	.	0	.	.	.
[Chair_DUM_0=.000]	-.543	.645	.708	1	.400	-1.807	.721
[Chair_DUM_0=1.000]	0[a]	.	.	0	.	.	.
[Chair_DUM_1=.000]	-.150	.424	.125	1	.724	-.981	.681
[Chair_DUM_1=1.000]	0[a]	.	.	0	.	.	.
[Chair_DUM_2=.000]	-.086	.442	.038	1	.846	-.952	.781
[Chair_DUM_2=1.000]	0[a]	.	.	0	.	.	.
[Chair_DUM_3=.000]	-.551	.449	1.505	1	.220	-1.431	.329
[Chair_DUM_3=1.000]	0[a]	.	.	0	.	.	.
[Chair_DUM_4=.000]	0[a]	.	.	0	.	.	.
[Chair_DUM_4=1.000]	0[a]	.	.	0	.	.	.
[SPPB_0=.000]	1.201	1.993	.363	1	.547	-2.705	5.108
[SPPB_0=1.000]	0[a]	.	.	0	.	.	.
[SPPB_1=.000]	3.734	1.279	8.517	1	.004	1.226	6.241
[SPPB_1=1.000]	0[a]	.	.	0	.	.	.
[SPPB_2=.000]	.590	1.008	.343	1	.558	-1.386	2.566

[SPPB_2=1.000]	0[a]	.	.	0	.	.	.
[SPPB_3=.000]	1.563	.848	3.395	1	.065	-.100	3.225
[SPPB_3=1.000]	0[a]	.	.	0	.	.	.
[SPPB_4=.000]	.672	.696	.931	1	.334	-.692	2.035
[SPPB_4=1.000]	0[a]	.	.	0	.	.	.
[SPPB_5=.000]	.260	.617	.177	1	.674	-.950	1.469
[SPPB_5=1.000]	0[a]	.	.	0	.	.	.
[SPPB_7=.000]	-1.090	.504	4.684	1	.030	-2.077	-.103
[SPPB_7=1.000]	0[a]	.	.	0	.	.	.
[SPPB_8=.000]	-.629	.507	1.543	1	.214	-1.623	.364
[SPPB_8=1.000]	0[a]	.	.	0	.	.	.
[SPPB_9=.000]	-.270	.475	.323	1	.570	-1.201	.661
[SPPB_9=1.000]	0[a]	.	.	0	.	.	.
[SPPB_10=.000]	.476	.577	.681	1	.409	-.655	1.607
[SPPB_10=1.000]	0[a]	.	.	0	.	.	.
[SPPB_11=.000]	.033	.648	.003	1	.959	-1.236	1.303
[SPPB_11=1.000]	0[a]	.	.	0	.	.	.
[SPPB_12=.000]	0[a]	.	.	0	.	.	.
[SPPB_12=1.000]	0[a]	.	.	0	.	.	.
[EQ5D_Mob_1=.000]	-.925	2.284	.164	1	.685	-5.402	3.552
[EQ5D_Mob_1=1.000]	0[a]	.	.	0	.	.	.
[EQ5D_Mob_2=.000]	-1.748	2.245	.606	1	.436	-6.147	2.652
[EQ5D_Mob_2=1.000]	0[a]	.	.	0	.	.	.
[EQ5D_Mob_3=.000]	0[a]	.	.	0	.	.	.
[EQ5D_Mob_3=1.000]	0[a]	.	.	0	.	.	.
[EQ5D_SC_1=.000]	-2.421	2.540	.909	1	.340	-7.399	2.556
[EQ5D_SC_1=1.000]	0[a]	.	.	0	.	.	.
[EQ5D_SC_2=.000]	-2.598	2.453	1.122	1	.289	-7.406	2.209
[EQ5D_SC_2=1.000]	0[a]	.	.	0	.	.	.
[EQ5D_SC_3=.000]	0[a]	.	.	0	.	.	.
[EQ5D_Usual_1=.000]	4.658	1.865	6.240	1	.012	1.003	8.312
[EQ5D_Usual_1=1.000]	0[a]	.	.	0	.	.	.
[EQ5D_Usual_2=.000]	3.098	1.788	3.003	1	.083	-.406	6.602
[EQ5D_Usual_2=1.000]	0[a]	.	.	0	.	.	.
[EQ5D_Usual_3=.000]	0[a]	.	.	0	.	.	.
[EQ5D_Usual_3=1.000]	0[a]	.	.	0	.	.	.
[EQ5D_Pain_1=.000]	.695	1.278	.296	1	.587	-1.810	3.199
[EQ5D_Pain_1=1.000]	0[a]	.	.	0	.	.	.
[EQ5D_Pain_2=.000]	.559	.932	.360	1	.548	-1.268	2.387
[EQ5D_Pain_2=1.000]	0[a]	.	.	0	.	.	.
[EQ5D_Pain_3=.000]	0[a]	.	.	0	.	.	.
[EQ5D_Pain_3=1.000]	0[a]	.	.	0	.	.	.
[EQ5D_Anx_1=.000]	.832	1.024	.661	1	.416	-1.175	2.839
[EQ5D_Anx_1=1.000]	0[a]	.	.	0	.	.	.
[EQ5D_Anx_2=.000]	.464	.806	.331	1	.565	-1.116	2.044
[EQ5D_Anx_2=1.000]	0[a]	.	.	0	.	.	.
[EQ5D_Anx_3=.000]	0[a]	.	.	0	.	.	.

	[EQ5D_Anx_3=1.000]	0[a]	.	.	0	.	.	.
	[w_FCI_0=.000]	.538	.784	.472	1	.492	-.998	2.074
	[w_FCI_0=1.000]	0[a]	.	.	0	.	.	.
	[w_FCI_1=.000]	-.015	.548	.001	1	.978	-1.089	1.059
	[w_FCI_1=1.000]	0[a]	.	.	0	.	.	.
	[w_FCI_2=.000]	.727	.415	3.075	1	.079	-.086	1.540
	[w_FCI_2=1.000]	0[a]	.	.	0	.	.	.
	[w_FCI_4=.000]	-.069	.417	.027	1	.868	-.886	.748
	[w_FCI_4=1.000]	0[a]	.	.	0	.	.	.
	[w_FCI_5=.000]	-.547	.444	1.520	1	.218	-1.417	.323
	[w_FCI_5=1.000]	0[a]	.	.	0	.	.	.
	[w_FCI_6=.000]	-.563	.496	1.289	1	.256	-1.535	.409
	[w_FCI_6=1.000]	0[a]	.	.	0	.	.	.
	[w_FCI_8=.000]	-1.173	.655	3.206	1	.073	-2.456	.111
	[w_FCI_8=1.000]	0[a]	.	.	0	.	.	.
	[w_FCI_9=.000]	-.913	.658	1.927	1	.165	-2.203	.376
	[w_FCI_9=1.000]	0[a]	.	.	0	.	.	.
	[w_FCI_10=.000]	-1.985	1.037	3.663	1	.056	-4.017	.048
	[w_FCI_10=1.000]	0[a]	.	.	0	.	.	.
	[w_FCI_11=.000]	-.233	1.155	.041	1	.840	-2.497	2.032
	[w_FCI_11=1.000]	0[a]	.	.	0	.	.	.
	[w_FCI_12=.000]	-1.149	1.269	.821	1	.365	-3.636	1.337
	[w_FCI_12=1.000]	0[a]	.	.	0	.	.	.
	[w_FCI_13=.000]	2.117	1.670	1.607	1	.205	-1.156	5.389
	[w_FCI_13=1.000]	0[a]	.	.	0	.	.	.
	[w_FCI_14=.000]	.994	1.992	.249	1	.618	-2.910	4.898
	[w_FCI_14=1.000]	0[a]	.	.	0	.	.	.
	[w_FCI_15=.000]	0[a]	.	.	0	.	.	.
	[w_FCI_16=.000]	0[a]	.	.	0	.	.	.
	[w_FCI_17=.000]	4.293	3.406	1.589	1	.208	-2.383	10.969
	[w_FCI_17=1.000]	0[a]	.	.	0	.	.	.
	[w_FCI_18=.000]	0[a]	.	.	0	.	.	.
	[w_FCI_18=1.000]	0[a]	.	.	0	.	.	.
	[w_FCI_19=.000]	0[a]	.	.	0	.	.	.
	[w_FCI_19=1.000]	0[a]	.	.	0	.	.	.
	[w_FCI_20=.000]	0[a]	.	.	0	.	.	.
	[w_FCI_21=.000]	0[a]	.	.	0	.	.	.
	[BBAQ_Time_1=.000]	.386	.829	.217	1	.641	-1.239	2.011
	[BBAQ_Time_1=1.000]	0[a]	.	.	0	.	.	.
	[BBAQ_Time_0=.000]	-.430	.797	.292	1	.589	-1.992	1.131
	[BBAQ_Time_0=1.000]	0[a]	.	.	0	.	.	.
	[BBAQ_Time_2=.000]	-.448	.809	.306	1	.580	-2.034	1.138
	[BBAQ_Time_2=1.000]	0[a]	.	.	0	.	.	.
	[BBAQ_Time_3=.000]	-1.077	.810	1.768	1	.184	-2.665	.511
	[BBAQ_Time_3=1.000]	0[a]	.	.	0	.	.	.
	[BBAQ_Time_4=.000]	-1.732	.841	4.243	1	.039	-3.380	-.084
	[BBAQ_Time_4=1.000]	0[a]	.	.	0	.	.	.

	[BBAQ_Time_5=.000]	-.945	.833	1.289	1	.256	-2.577	.687
	[BBAQ_Time_5=1.000]	0[a]	.	.	0	.	.	.
	[BBAQ_Time_6=.000]	-.018	.745	.001	1	.980	-1.478	1.442
	[BBAQ_Time_6=1.000]	0[a]	.	.	0	.	.	.
	[BBAQ_Time_7=.000]	-.712	.937	.578	1	.447	-2.548	1.123
	[BBAQ_Time_7=1.000]	0[a]	.	.	0	.	.	.
	[BBAQ_Time_8=.000]	1.332	1.245	1.145	1	.285	-1.108	3.772
	[BBAQ_Time_8=1.000]	0[a]	.	.	0	.	.	.
	[BBAQ_Time_9=.000]	0[a]	.	.	0	.	.	.
	[BBAQ_Time_9=1.000]	0[a]	.	.	0	.	.	.
	[BBAQ_Social_0=.000]	.933	.727	1.647	1	.199	-.492	2.357
	[BBAQ_Social_0=1.000]	0[a]	.	.	0	.	.	.
	[BBAQ_Social_1=.000]	1.283	.769	2.782	1	.095	-.225	2.791
	[BBAQ_Social_1=1.000]	0[a]	.	.	0	.	.	.
	[BBAQ_Social_2=.000]	.991	.766	1.674	1	.196	-.510	2.493
	[BBAQ_Social_2=1.000]	0[a]	.	.	0	.	.	.
	[BBAQ_Social_3=.000]	.705	.675	1.089	1	.297	-.619	2.028
	[BBAQ_Social_3=1.000]	0[a]	.	.	0	.	.	.
	[BBAQ_Social_4=.000]	1.416	.785	3.257	1	.071	-.122	2.954
	[BBAQ_Social_4=1.000]	0[a]	.	.	0	.	.	.
	[BBAQ_Social_5=.000]	.389	.838	.216	1	.642	-1.253	2.031
	[BBAQ_Social_5=1.000]	0[a]	.	.	0	.	.	.
	[BBAQ_Social_6=.000]	.502	.661	.576	1	.448	-.793	1.797
	[BBAQ_Social_6=1.000]	0[a]	.	.	0	.	.	.
	[BBAQ_Social_7=.000]	-.575	1.203	.229	1	.632	-2.933	1.782
	[BBAQ_Social_7=1.000]	0[a]	.	.	0	.	.	.
	[BBAQ_Social_8=.000]	1.120	1.014	1.221	1	.269	-.867	3.107
	[BBAQ_Social_8=1.000]	0[a]	.	.	0	.	.	.
	[BBAQ_Social_9=.000]	0[a]	.	.	0	.	.	.
	[BBAQ_Social_9=1.000]	0[a]	.	.	0	.	.	.
	[BBAQ_Energy_0=.000]	1.170	.488	5.752	1	.016	.214	2.125
	[BBAQ_Energy_0=1.000]	0[a]	.	.	0	.	.	.
	[BBAQ_Energy_1=.000]	1.048	.551	3.619	1	.057	-.032	2.129
	[BBAQ_Energy_1=1.000]	0[a]	.	.	0	.	.	.
	[BBAQ_Energy_2=.000]	.569	.508	1.254	1	.263	-.427	1.565
	[BBAQ_Energy_2=1.000]	0[a]	.	.	0	.	.	.
	[BBAQ_Energy_3=.000]	1.139	.475	5.761	1	.016	.209	2.070
	[BBAQ_Energy_3=1.000]	0[a]	.	.	0	.	.	.
	[BBAQ_Energy_4=.000]	.483	.503	.922	1	.337	-.503	1.468
	[BBAQ_Energy_4=1.000]	0[a]	.	.	0	.	.	.
	[BBAQ_Energy_7=.000]	.822	.822	1.002	1	.317	-.788	2.433
	[BBAQ_Energy_7=1.000]	0[a]	.	.	0	.	.	.
	[BBAQ_Energy_8=.000]	-1.901	1.268	2.246	1	.134	-4.387	.585
	[BBAQ_Energy_8=1.000]	0[a]	.	.	0	.	.	.
	[BBAQ_Energy_9=.000]	.391	.741	.278	1	.598	-1.062	1.844
	[BBAQ_Energy_9=1.000]	0[a]	.	.	0	.	.	.
	[BBAQ_Will_0=.000]	.324	.669	.235	1	.628	-.987	1.636
	[BBAQ_Will_0=1.000]	0[a]	.	.	0	.	.	.

	[BBAQ_Will_1=.000]	.211	.735	.082	1	.774	-1.230	1.651
	[BBAQ_Will_1=1.000]	0[a]	.	.	0	.	.	.
	[BBAQ_Will_2=.000]	-.211	.691	.093	1	.760	-1.565	1.144
	[BBAQ_Will_2=1.000]	0[a]	.	.	0	.	.	.
	[BBAQ_Will_3=.000]	-.358	.673	.283	1	.594	-1.677	.960
	[BBAQ_Will_3=1.000]	0[a]	.	.	0	.	.	.
	[BBAQ_Will_4=.000]	.404	.692	.341	1	.559	-.952	1.760
	[BBAQ_Will_4=1.000]	0[a]	.	.	0	.	.	.
	[BBAQ_Will_5=.000]	.961	.667	2.076	1	.150	-.346	2.267
	[BBAQ_Will_5=1.000]	0[a]	.	.	0	.	.	.
	[BBAQ_Will_6=.000]	.669	.642	1.086	1	.297	-.590	1.928
	[BBAQ_Will_6=1.000]	0[a]	.	.	0	.	.	.
	[BBAQ_Will_7=.000]	.956	.730	1.718	1	.190	-.474	2.386
	[BBAQ_Will_7=1.000]	0[a]	.	.	0	.	.	.
	[BBAQ_Will_8=.000]	-.293	.786	.139	1	.709	-1.834	1.247
	[BBAQ_Will_8=1.000]	0[a]	.	.	0	.	.	.
	[BBAQ_Will_9=.000]	0[a]	.	.	0	.	.	.
	[BBAQ_Will_9=1.000]	0[a]	.	.	0	.	.	.
	[BBAQ_Injury_0=.000]	-.639	.904	.500	1	.479	-2.411	1.132
	[BBAQ_Injury_0=1.000]	0[a]	.	.	0	.	.	.
	[BBAQ_Injury_1=.000]	-.900	.948	.902	1	.342	-2.758	.957
	[BBAQ_Injury_1=1.000]	0[a]	.	.	0	.	.	.
	[BBAQ_Injury_2=.000]	-.967	.947	1.042	1	.307	-2.823	.889
	[BBAQ_Injury_2=1.000]	0[a]	.	.	0	.	.	.
	[BBAQ_Injury_3=.000]	-.954	.888	1.153	1	.283	-2.695	.787
	[BBAQ_Injury_3=1.000]	0[a]	.	.	0	.	.	.
	[BBAQ_Injury_4=.000]	-.677	.942	.516	1	.473	-2.523	1.170
	[BBAQ_Injury_4=1.000]	0[a]	.	.	0	.	.	.
	[BBAQ_Injury_5=.000]	-1.422	.971	2.147	1	.143	-3.325	.480
	[BBAQ_Injury_5=1.000]	0[a]	.	.	0	.	.	.
	[BBAQ_Injury_6=.000]	-.934	.890	1.101	1	.294	-2.678	.811
	[BBAQ_Injury_6=1.000]	0[a]	.	.	0	.	.	.
	[BBAQ_Injury_7=.000]	-1.029	1.056	.949	1	.330	-3.099	1.041
	[BBAQ_Injury_7=1.000]	0[a]	.	.	0	.	.	.
	[BBAQ_Injury_8=.000]	-3.306	1.079	9.397	1	.002	-5.421	-1.192
	[BBAQ_Injury_8=1.000]	0[a]	.	.	0	.	.	.
	[BBAQ_Injury_9=.000]	0[a]	.	.	0	.	.	.
	[BBAQ_Injury_9=1.000]	0[a]	.	.	0	.	.	.
	[BBAQ_Skill_0=.000]	.902	.789	1.306	1	.253	-.645	2.449
	[BBAQ_Skill_0=1.000]	0[a]	.	.	0	.	.	.
	[BBAQ_Skill_1=.000]	.522	.785	.441	1	.50\\’’’’’’’’’\ 7	-1.018	2.061
	[BBAQ_Skill_1=1.000]	0[a]	.	.	0	.	.	.
	[BBAQ_Skill_2=.000]	.528	.786	.452	1	.501	-1.012	2.068
	[BBAQ_Skill_2=1.000]	0[a]	.	.	0	.	.	.
	[BBAQ_Skill_3=.000]	1.148	.783	2.146	1	.143	-.388	2.683
	[BBAQ_Skill_3=1.000]	0[a]	.	.	0	.	.	.
	[BBAQ_Skill_4=.000]	.582	.819	.506	1	.477	-1.022	2.187

[BBAQ_Skill_4=1.000]	0[a]	.	.	0	.	.	.
[BBAQ_Skill_5=.000]	1.234	.820	2.267	1	.132	-.372	2.840
[BBAQ_Skill_5=1.000]	0[a]	.	.	0	.	.	.
[BBAQ_Skill_6=.000]	.126	.787	.025	1	.873	-1.417	1.668
[BBAQ_Skill_6=1.000]	0[a]	.	.	0	.	.	.
[BBAQ_Skill_7=.000]	2.181	.952	5.247	1	.022	.315	4.047
[BBAQ_Skill_7=1.000]	0[a]	.	.	0	.	.	.
[BBAQ_Skill_8=.000]	-.904	.886	1.039	1	.308	-2.641	.834
[BBAQ_Skill_8=1.000]	0[a]	.	.	0	.	.	.
[BBAQ_Skill_9=.000]	0[a]	.	.	0	.	.	.
[BBAQ_Skill_9=1.000]	0[a]	.	.	0	.	.	.
[BBAQ_Resou_0=.000]	-.143	.525	.074	1	.785	-1.171	.885
[BBAQ_Resou_0=1.000]	0[a]	.	.	0	.	.	.
[BBAQ_Resou_1=.000]	.119	.575	.043	1	.836	-1.008	1.245
[BBAQ_Resou_1=1.000]	0[a]	.	.	0	.	.	.
[BBAQ_Resou_2=.000]	-.132	.536	.061	1	.805	-1.182	.918
[BBAQ_Resou_2=1.000]	0[a]	.	.	0	.	.	.
[BBAQ_Resou_3=.000]	.323	.468	.477	1	.490	-.594	1.240
[BBAQ_Resou_3=1.000]	0[a]	.	.	0	.	.	.
[BBAQ_Resou_4=.000]	-.305	.511	.357	1	.550	-1.306	.696
[BBAQ_Resou_4=1.000]	0[a]	.	.	0	.	.	.
[BBAQ_Resou_5=.000]	.020	.547	.001	1	.971	-1.052	1.092
[BBAQ_Resou_5=1.000]	0[a]	.	.	0	.	.	.
[BBAQ_Resou_6=.000]	.478	.425	1.266	1	.261	-.355	1.312
[BBAQ_Resou_6=1.000]	0[a]	.	.	0	.	.	.
[BBAQ_Resou_7=.000]	.978	.591	2.740	1	.098	-.180	2.135
[BBAQ_Resou_7=1.000]	0[a]	.	.	0	.	.	.
[BBAQ_Resou_8=.000]	.501	.601	.695	1	.404	-.677	1.678
[BBAQ_Resou_8=1.000]	0[a]	.	.	0	.	.	.
[BBAQ_Resou_9=.000]	0[a]	.	.	0	.	.	.
[BBAQ_Resou_9=1.000]	0[a]	.	.	0	.	.	.
[SPPB_6=.000]	0[a]	.	.	0	.	.	.
[SPPB_6=1.000]	0[a]	.	.	0	.	.	.

Link function: Logit.
